# Post-acute COVID-19 neuropsychiatric symptoms are not associated with ongoing nervous system injury

**DOI:** 10.1093/braincomms/fcad357

**Published:** 2023-12-27

**Authors:** Maxime Taquet, Zuzanna Skorniewska, Henrik Zetterberg, John R Geddes, Catherine J Mummery, James D Chalmers, Ling-Pei Ho, Alex Horsley, Michael Marks, Krisnah Poinasamy, Betty Raman, Olivia C Leavy, Matthew Richardson, Omer Elneima, Hamish J C McAuley, Aarti Shikotra, Amisha Singapuri, Marco Sereno, Ruth M Saunders, Victoria Claire Harris, Linzy Houchen-Wolloff, Parisa Mansoori, Neil J Greening, Ewen M Harrison, Annemarie B Docherty, Nazir I Lone, Jennifer Quint, William Greenhalf, Louise V Wain, Christopher E Brightling, Rachael E Evans, Paul J Harrison, Ivan Koychev, PHOSP-COVID Study Collaborative Group, C E Brightling, R A Evans, L V Wain, J D Chalmers, V C Harris, L P Ho, A Horsley, M Marks, K Poinasamy, B Raman, A Shikotra, A Singapuri, C E Brightling, R A Evans, L V Wain, R Dowling, C Edwardson, O Elneima, S Finney, N J Greening, B Hargadon, V Harris, L Houchen--Wolloff, O C Leavy, H J C McAuley, C Overton, T Plekhanova, R M Saunders, M Sereno, A Singapuri, A Shikotra, C Taylor, S Terry, C Tong, B Zhao, D Lomas, E Sapey, C Berry, C E Bolton, N Brunskill, E R Chilvers, R Djukanovic, Y Ellis, D Forton, N French, J George, N A Hanley, N Hart, L McGarvey, N Maskell, H McShane, M Parkes, D Peckham, P Pfeffer, A Sayer, A Sheikh, A A R Thompson, N Williams, C E Brightling, W Greenhalf, M G Semple, M Ashworth, H E Hardwick, L Lavelle-Langham, W Reynolds, M Sereno, R M Saunders, A Singapuri, V Shaw, A Shikotra, B Venson, L V Wain, A B Docherty, E M Harrison, A Sheikh, J K Baillie, C E Brightling, L Daines, R Free, R A Evans, S Kerr, O C Leavy, N I Lone, H J C McAuley, R Pius, J Quint, M Richardson, M Sereno, M Thorpe, L V Wain, M Halling-Brown, F Gleeson, J Jacob, S Neubauer, B Raman, S Siddiqui, J M Wild, S Aslani, P Jezzard, H Lamlum, W Lilaonitkul, E Tunnicliffe, J Willoughby, L V Wain, J K Baillie, H Baxendale, C E Brightling, M Brown, J D Chalmers, R A Evans, B Gooptu, W Greenhalf, H E Hardwick, R G Jenkins, D Jones, I Koychev, C Langenberg, A Lawrie, P L Molyneaux, A Shikotra, J Pearl, M Ralser, N Sattar, R M Saunders, J T Scott, T Shaw, D Thomas, D Wilkinson, L G Heaney, A De Soyza, D Adeloye, C E Brightling, J S Brown, J Busby, J D Chalmers, C Echevarria, L Daines, O Elneima, R A Evans, J Hurst, P Novotny, P Pfeffer, K Poinasamy, J Quint, I Rudan, E Sapey, M Shankar-Hari, A Sheikh, S Siddiqui, S Walker, B Zheng, J R Geddes, M Hotopf, K Abel, R Ahmed, L Allan, C Armour, D Baguley, D Baldwin, C Ballard, K Bhui, G Breen, M Broome, T Brugha, E Bullmore, D Burn, F Callard, J Cavanagh, T Chalder, D Clark, A David, B Deakin, H Dobson, B Elliott, J Evans, R Francis, E Guthrie, P Harrison, M Henderson, A Hosseini, N Huneke, M Husain, T Jackson, I Jones, T Kabir, P Kitterick, A Korszun, I Koychev, J Kwan, A Lingford-Hughes, P Mansoori, H McAllister-Williams, K McIvor, L Milligan, R Morriss, E Mukaetova-Ladinska, K Munro, A Nevado-Holgado, T Nicholson, S Paddick, C Pariante, J Pimm, K Saunders, M Sharpe, G Simons, R Upthegrove, S Wessely, G P McCann, S Amoils, C Antoniades, A Banerjee, R Bell, A Bularga, C Berry, P Chowienczyk, J P Greenwood, A D Hughes, K Khunti, L Kingham, C Lawson, K Mangion, N L Mills, A J Moss, S Neubauer, B Raman, A N Sattar, C L Sudlow, M Toshner, P J M Openshaw, D Altmann, J K Baillie, R Batterham, H Baxendale, N Bishop, C E Brightling, P C Calder, R A Evans, J L Heeney, T Hussell, P Klenerman, J M Lord, P Moss, S L Rowland-Jones, W Schwaeble, M G Semple, R S Thwaites, L Turtle, L V Wain, S Walmsley, D Wraith, M J Rowland, A Rostron, J K Baillie, B Connolly, A B Docherty, N I Lone, D F McAuley, D Parekh, A Rostron, J Simpson, C Summers, R G Jenkins, J Porter, R J Allen, R Aul, J K Baillie, S Barratt, P Beirne, J Blaikley, R C Chambers, N Chaudhuri, C Coleman, E Denneny, L Fabbri, P M George, M Gibbons, F Gleeson, B Gooptu, B Guillen Guio, I Hall, N A Hanley, L P Ho, E Hufton, J Jacob, I Jarrold, G Jenkins, S Johnson, M G Jones, S Jones, F Khan, P Mehta, J Mitchell, P L Molyneaux, J E Pearl, K Piper Hanley, K Poinasamy, J Quint, D Parekh, P Rivera-Ortega, L C Saunders, M G Semple, J Simpson, D Smith, M Spears, L G Spencer, S Stanel, I Stewart, A A R Thompson, D Thickett, R Thwaites, L V Wain, S Walker, S Walsh, J M Wild, D G Wootton, L Wright, S Heller, M J Davies, H Atkins, S Bain, J Dennis, K Ismail, D Johnston, P Kar, K Khunti, C Langenberg, P McArdle, A McGovern, T Peto, J Petrie, E Robertson, N Sattar, K Shah, J Valabhji, B Young, L S Howard, Mark Toshner, C Berry, P Chowienczyk, D Lasserson, A Lawrie, O C Leavy, J Mitchell, L Price, J Quint, J Rossdale, N Sattar, C Sudlow, A A R Thompson, J M Wild, M Wilkins, S J Singh, W D-C Man, J M Lord, N J Greening, T Chalder, J T Scott, N Armstrong, E Baldry, M Baldwin, N Basu, M Beadsworth, L Bishop, C E Bolton, A Briggs, M Buch, G Carson, J Cavanagh, H Chinoy, E Daynes, S Defres, R A Evans, P Greenhaff, S Greenwood, M Harvie, M Husain, S MacDonald, A McArdle, H J C McAuley, A McMahon, M McNarry, C Nolan, K O'Donnell, D Parekh, J Pimm, J Sargent, L Sigfrid, M Steiner, D Stensel, A L Tan, J Whitney, D Wilkinson, D Wilson, M Witham, D G Wootton, T Yates, D Thomas, N Brunskill, S Francis, S Greenwood, C Laing, K Bramham, P Chowdhury, A Frankel, L Lightstone, S McAdoo, K McCafferty, M Ostermann, N Selby, C Sharpe, M Willicombe, A Shaw, L Armstrong, B Hairsine, H Henson, C Kurasz, L Shenton, S Fairbairn, A Dell, N Hawkings, J Haworth, M Hoare, A Lucey, V Lewis, G Mallison, H Nassa, C Pennington, A Price, C Price, A Storrie, G Willis, S Young, P Pfeffer, K Chong-James, C David, W Y James, A Martineau, O Zongo, A Sanderson, L G Heaney, C Armour, V Brown, T Craig, S Drain, B King, N Magee, D McAulay, E Major, L McGarvey, J McGinness, R Stone, A Haggar, A Bolger, F Davies, J Lewis, A Lloyd, R Manley, E McIvor, D Menzies, K Roberts, W Saxon, D Southern, C Subbe, V Whitehead, H El-Taweel, J Dawson, L Robinson, D Saralaya, L Brear, K Regan, K Storton, J Fuld, A Bermperi, I Cruz, K Dempsey, A Elmer, H Jones, S Jose, S Marciniak, M Parkes, C Ribeiro, J Taylor, M Toshner, L Watson, J Worsley, R Sabit, L Broad, A Buttress, T Evans, M Haynes, L Jones, L Knibbs, A McQueen, C Oliver, K Paradowski, J Williams, E Harris, C Sampson, C Lynch, E Davies, C Evenden, A Hancock, K Hancock, M Rees, L Roche, N Stroud, T Thomas-Woods, M Babores, J Bradley-Potts, M Holland, N Keenan, S Shashaa, H Wassall, E Beranova, H Weston, T Cosier, L Austin, J Deery, T Hazelton, C Price, H Ramos, R Solly, S Turney, L Pearce, W McCormack, S Pugmire, W Stoker, A Wilson, N Hart, L A Aguilar Jimenez, G Arbane, S Betts, K Bisnauthsing, A Dewar, P Chowdhury, A Dewar, G Kaltsakas, H Kerslake, M M Magtoto, P Marino, L M Martinez, M Ostermann, J Rossdale, T S Solano, E Wynn, N Williams, W Storrar, M Alvarez Corral, A Arias, E Bevan, D Griffin, J Martin, J Owen, S Payne, A Prabhu, A Reed, C Wrey Brown, C Lawson, T Burdett, J Featherstone, A Layton, C Mills, L Stephenson, N Easom, P Atkin, K Brindle, M G Crooks, K Drury, R Flockton, L Holdsworth, A Richards, D L Sykes, S Thackray-Nocera, C Wright, K E Lewis, A Mohamed, G Ross, S Coetzee, K Davies, R Hughes, R Loosley, L O'Brien, Z Omar, H McGuinness, E Perkins, J Phipps, A Taylor, H Tench, R Wolf-Roberts, L S Howard, O Kon, D C Thomas, S Anifowose, L Burden, E Calvelo, B Card, C Carr, E R Chilvers, D Copeland, P Cullinan, P Daly, L Evison, T Fayzan, H Gordon, S Haq, R G Jenkins, C King, K March, M Mariveles, L McLeavey, N Mohamed, S Moriera, U Munawar, J Nunag, U Nwanguma, L Orriss-Dib, A Ross, M Roy, E Russell, K Samuel, J Schronce, N Simpson, L Tarusan, C Wood, N Yasmin, R Reddy, A-M Guerdette, M Hewitt, K Warwick, S White, A M Shah, C J Jolley, O Adeyemi, R Adrego, H Assefa-Kebede, J Breeze, M Brown, S Byrne, T Chalder, P Dulawan, N Hart, A Hayday, A Hoare, A Knighton, M Malim, S Patale, I Peralta, N Powell, A Ramos, K Shevket, F Speranza, A Te, P Beirne, A Ashworth, J Clarke, C Coupland, M Dalton, E Wade, C Favager, J Greenwood, J Glossop, L Hall, T Hardy, A Humphries, J Murira, D Peckham, S Plein, J Rangeley, G Saalmink, A L Tan, B Whittam, N Window, J Woods, G Coakley, D G Wootton, L Turtle, L Allerton, A M All, M Beadsworth, A Berridge, J Brown, S Cooper, A Cross, S Defres, S L Dobson, J Earley, N French, W Greenhalf, H E Hardwick, K Hainey, J Hawkes, V Highett, S Kaprowska, A L Key, L Lavelle-Langham, N Lewis-Burke, G Madzamba, F Malein, S Marsh, C Mears, L Melling, M J Noonan, L Poll, J Pratt, E Richardson, A Rowe, M G Semple, V Shaw, K A Tripp, L O Wajero, S A Williams-Howard, J Wyles, S N Diwanji, P Papineni, S Gurram, S Quaid, G F Tiongson, E Watson, B Al-Sheklly, A Horsley, C Avram, J Blaikely, M Buch, N Choudhury, D Faluyi, T Felton, T Gorsuch, N A Hanley, T Hussell, Z Kausar, N Odell, R Osbourne, K Piper Hanley, K Radhakrishnan, S Stockdale, A De Soyza, C Echevarria, A Ayoub, J Brown, G Burns, G Davies, H Fisher, C Francis, A Greenhalgh, P Hogarth, J Hughes, K Jiwa, G Jones, G MacGowan, D Price, A Sayer, J Simpson, H Tedd, S Thomas, S West, M Witham, S Wright, A Young, M J McMahon, P Neill, D Anderson, H Bayes, C Berry, D Grieve, I B McInnes, N Basu, A Brown, A Dougherty, K Fallon, L Gilmour, K Mangion, A Morrow, K Scott, R Sykes, E K Sage, F Barrett, A Donaldson, M Patel, D Bell, A Brown, M Brown, R Hamil, K Leitch, L Macliver, J Quigley, A Smith, B Welsh, G Choudhury, J K Baillie, S Clohisey, A Deans, A B Docherty, J Furniss, E M Harrison, S Kelly, N I Lone, A Sheikh, J D Chalmers, D Connell, A Elliott, C Deas, J George, S Mohammed, J Rowland, A R Solstice, D Sutherland, C J Tee, B Jayaraman, T Light, C E Bolton, P Almeida, J Bonnington, M Chrystal, C Dupont, P Greenhaff, A Gupta, L Howard, W Jang, S Linford, L Matthews, R Needham, A Nikolaidis, S Prosper, K Shaw, A K Thomas, L P Ho, N M Rahman, M Ainsworth, A Alamoudi, A Bates, A Bloss, A Burns, P Carter, J Chen, F Conneh, T Dong, R I Evans, E Fraser, X Fu, J R Geddes, F Gleeson, P Harrison, M Havinden-Williams, P Jezzard, N Kanellakis, I Koychev, P Kurupati, X Li, H McShane, C Megson, K Motohashi, S Neubauer, D Nicoll, G Ogg, E Pacpaco, M Pavlides, Y Peng, N Petousi, N Rahman, B Raman, M J Rowland, K Saunders, M Sharpe, N Talbot, E Tunnicliffe, W D-C Man, B Patel, R E Barker, D Cristiano, N Dormand, M Gummadi, S Kon, K Liyanage, C M Nolan, S Patel, O Polgar, P Shah, S J Singh, J A Walsh, J Hurst, H Jarvis, S Mandal, S Ahmad, S Brill, L Lim, D Matila, O Olaosebikan, C Singh, M Toshner, H Baxendale, L Garner, C Johnson, J Mackie, A Michael, J Pack, K Paques, H Parfrey, J Parmar, N Diar Bakerly, P Dark, D Evans, E Hardy, A Harvey, D Holgate, S Knight, N Mairs, N Majeed, L McMorrow, J Oxton, J Pendlebury, C Summersgill, R Ugwuoke, S Whittaker, W Matimba-Mupaya, S Strong-Sheldrake, S L Rowland-Jones, A A R Thompson, J Bagshaw, M Begum, K Birchall, R Butcher, H Carborn, F Chan, K Chapman, Y Cheng, L Chetham, C Clark, Z Coburn, J Cole, M Dixon, A Fairman, J Finnigan, H Foot, D Foote, A Ford, R Gregory, K Harrington, L Haslam, L Hesselden, J Hockridge, A Holbourn, B Holroyd-Hind, L Holt, A Howell, E Hurditch, F Ilyas, C Jarman, A Lawrie, E Lee, J-H Lee, R Lenagh, A Lye, I Macharia, M Marshall, A Mbuyisa, J McNeill, S Megson, J Meiring, L Milner, S Misra, H Newell, T Newman, C Norman, L Nwafor, D Pattenadk, M Plowright, J Porter, P Ravencroft, C Roddis, J Rodger, P Saunders, J Sidebottom, J Smith, L Smith, N Steele, G Stephens, R Stimpson, B Thamu, N Tinker, K Turner, H Turton, P Wade, S Walker, J Watson, I Wilson, A Zawia, R Aul, M Ali, A Dunleavy, D Forton, N Msimanga, M Mencias, T Samakomva, S Siddique, J Teixeira, V Tavoukjian, J Hutchinson, L Allsop, K Bennett, P Buckley, M Flynn, M Gill, C Goodwin, M Greatorex, H Gregory, C Heeley, L Holloway, M Holmes, J Kirk, W Lovegrove, T A Sewell, S Shelton, D Sissons, K Slack, S Smith, D Sowter, S Turner, V Whitworth, I Wynter, L Warburton, S Painter, J Tomlinson, C Vickers, T Wainwright, D Redwood, J Tilley, S Palmer, G A Davies, L Connor, A Cook, T Rees, F Thaivalappil, C Thomas, A Butt, M Coulding, H Jones, S Kilroy, J McCormick, J McIntosh, H Savill, V Turner, J Vere, E Fraile, J Ugoji, S S Kon, H Lota, G Landers, M Nasseri, S Portukhay, A Hormis, A Daniels, J Ingham, L Zeidan, M Chablani, L Osborne, M Marks, J S Brown, N Ahwireng, B Bang, D Basire, R C Chambers, A Checkley, R Evans, M Heightman, T Hillman, J Hurst, J Jacob, S Janes, R Jastrub, M Lipman, S Logan, D Lomas, M Merida Morillas, H Plant, J C Porter, K Roy, E Wall, D Parekh, N Ahmad Haider, C Atkin, R Baggott, M Bates, A Botkai, A Casey, B Cooper, J Dasgin, K Draxlbauer, N Gautam, J Hazeldine, T Hiwot, S Holden, K Isaacs, T Jackson, S Johnson, V Kamwa, D Lewis, J M Lord, S Madathil, C McGhee, K Mcgee, A Neal, A Newton Cox, J Nyaboko, D Parekh, Z Peterkin, H Qureshi, L Ratcliffe, E Sapey, J Short, T Soulsby, J Stockley, Z Suleiman, T Thompson, M Ventura, S Walder, C Welch, D Wilson, S Yasmin, K P Yip, P Beckett, C Dickens, U Nanda, C E Brightling, R A Evans, M Aljaroof, N Armstrong, H Arnold, H Aung, M Bakali, M Bakau, M Baldwin, M Bingham, M Bourne, C Bourne, N Brunskill, P Cairns, L Carr, A Charalambou, C Christie, M J Davies, S Diver, S Edwards, C Edwardson, O Elneima, H Evans, J Finch, S Glover, N Goodman, B Gootpu, N J Greening, K Hadley, P Haldar, B Hargadon, V C Harris, L Houchen-Wolloff, W Ibrahim, L Ingram, K Khunti, A Lea, D Lee, G P McCann, H J C McAuley, P McCourt, T Mcnally, A Moss, W Monteiro, M Pareek, S Parker, A Rowland, A Prickett, I N Qureshi, R Russell, M Sereno, A Shikotra, S Siddiqui, A Singapuri, S J Singh, J Skeemer, M Soares, E Stringer, T Thornton, M Tobin, L V Wain, T J C Ward, F Woodhead, T Yates, A Yousuf, M G Jones, C Childs, R Djukanovic, S Fletcher, M Harvey, E Marouzet, B Marshall, R Samuel, T Sass, T Wallis, H Wheeler, R Dharmagunawardena, E Bright, P Crisp, M Stern, A Wight, L Bailey, A Reddington, A Ashish, J Cooper, E Robinson, A Broadley, K Howard, L Barman, C Brookes, K Elliott, L Griffiths, Z Guy, D Ionita, H Redfearn, C Sarginson, A Turnbull, Y Ellis, M Marks, A Briggs, K Holmes, Asthma UK, British Lung, K Poinasamy, S Walker, M Halling-Brown, G Breen, M Hotopf, K Lewis, N Williams

**Affiliations:** Department of Psychiatry, University of Oxford, Oxford OX3 7JX, UK; Oxford Health NHS Foundation Trust, Oxford OX3 7JX, UK; Department of Psychiatry, University of Oxford, Oxford OX3 7JX, UK; Department of Psychiatry and Neurochemistry, Institute of Neuroscience and Physiology, The Sahlgrenska Academy at the University of Gothenburg, Mölndal 413 90, Sweden; Clinical Neurochemistry Laboratory, Sahlgrenska University Hospital, Mölndal 413 90, Sweden; Department of Neurodegenerative Disease, UCL Institute of Neurology, London WC1N 3BG, UK; UK Dementia Research Institute at UCL, London WC1N 3BG, UK; Hong Kong Center for Neurodegenerative Diseases, Hong Kong, China; Wisconsin Alzheimer’s Disease Research Center, University of Wisconsin School of Medicine and Public Health, University of Wisconsin-Madison, Madison, WI 53792, USA; Department of Psychiatry, University of Oxford, Oxford OX3 7JX, UK; Oxford Health NHS Foundation Trust, Oxford OX3 7JX, UK; Department of Neurodegenerative Disease, UCL Institute of Neurology, London WC1N 3BG, UK; University of Dundee, Ninewells Hospital and Medical School, Dundee DD1 9SY, UK; MRC Human Immunology Unit, University of Oxford, Oxford OX3 9DS, UK; Division of Infection, Immunity & Respiratory Medicine, Faculty of Biology, Medicine and Health, University of Manchester, Manchester M13 9PL, UK; Manchester University NHS Foundation Trust, Manchester M13 9WL, UK; Department of Clinical Research, London School of Hygiene & Tropical Medicine, London WC1E 7HT, UK; Hospital for Tropical Diseases, University College London Hospital, London WC1E 6JD, UK; Division of Infection and Immunity, University College London, London WC1E 6BT, UK; Asthma and Lung UK, London E1 8AA, UK; Radcliffe Department of Medicine, University of Oxford, Oxford OX3 9DU, UK; Oxford University Hospitals NHS Foundation Trust, Oxford OX3 9DU, UK; Department of Population Health Sciences, University of Leicester, Leicester LE1 7RH, UK; The Institute for Lung Health, NIHR Leicester Biomedical Research Centre, University of Leicester, Leicester LE3 9QP, UK; The Institute for Lung Health, NIHR Leicester Biomedical Research Centre, University of Leicester, Leicester LE3 9QP, UK; The Institute for Lung Health, NIHR Leicester Biomedical Research Centre, University of Leicester, Leicester LE3 9QP, UK; NIHR Leicester Biomedical Research Centre, University of Leicester, Leicester LE5 4PW, UK; The Institute for Lung Health, NIHR Leicester Biomedical Research Centre, University of Leicester, Leicester LE3 9QP, UK; The Institute for Lung Health, NIHR Leicester Biomedical Research Centre, University of Leicester, Leicester LE3 9QP, UK; The Institute for Lung Health, NIHR Leicester Biomedical Research Centre, University of Leicester, Leicester LE3 9QP, UK; The Institute for Lung Health, NIHR Leicester Biomedical Research Centre, University of Leicester, Leicester LE3 9QP, UK; University Hospitals of Leicester NHS Trust, Leicester LE5 4PW, UK; Centre for Exercise and Rehabilitation Science, NIHR Leicester Biomedical Research Centre-Respiratory, University of Leicester, Leicester LE5 4PW, UK; Department of Respiratory Sciences, University of Leicester, Leicester LE1 9HN, UK; Therapy Department, University Hospitals of Leicester, NHS Trust, Leicester LE5 4PW, UK; MQ: Transforming Mental Health, London EC1Y 0TH, UK; The Institute for Lung Health, NIHR Leicester Biomedical Research Centre, University of Leicester, Leicester LE3 9QP, UK; Centre for Medical Informatics, The Usher Institute, University of Edinburgh, Edinburgh EH16 4SS, UK; Centre for Medical Informatics, The Usher Institute, University of Edinburgh, Edinburgh EH16 4SS, UK; Usher Institute, University of Edinburgh, Edinburgh EH16 4SS, UK; Royal Infirmary of Edinburgh, NHS Lothian, Edinburgh EH16 4SA, UK; National Heart and Lung Institute, Imperial College London, London SW3 6LY, UK; University of Liverpool, Liverpool L69 3BX, UK; The CRUK Liverpool Experimental Cancer Medicine Centre, Liverpool L69 3GL, UK; Liverpool University Hospitals NHS Foundation Trust, Liverpool L7 8YE, UK; Department of Population Health Sciences, University of Leicester, Leicester LE1 7RH, UK; The Institute for Lung Health, NIHR Leicester Biomedical Research Centre, University of Leicester, Leicester LE3 9QP, UK; The Institute for Lung Health, NIHR Leicester Biomedical Research Centre, University of Leicester, Leicester LE3 9QP, UK; The Institute for Lung Health, NIHR Leicester Biomedical Research Centre, University of Leicester, Leicester LE3 9QP, UK; University Hospitals of Leicester NHS Trust, Leicester LE5 4PW, UK; Department of Psychiatry, University of Oxford, Oxford OX3 7JX, UK; Oxford Health NHS Foundation Trust, Oxford OX3 7JX, UK; Department of Psychiatry, University of Oxford, Oxford OX3 7JX, UK

**Keywords:** neural injury, long COVID, biomarkers

## Abstract

A proportion of patients infected with severe acute respiratory syndrome coronavirus 2 experience a range of neuropsychiatric symptoms months after infection, including cognitive deficits, depression and anxiety. The mechanisms underpinning such symptoms remain elusive. Recent research has demonstrated that nervous system injury can occur during COVID-19. Whether ongoing neural injury in the months after COVID-19 accounts for the ongoing or emergent neuropsychiatric symptoms is unclear. Within a large prospective cohort study of adult survivors who were hospitalized for severe acute respiratory syndrome coronavirus 2 infection, we analysed plasma markers of nervous system injury and astrocytic activation, measured 6 months post-infection: neurofilament light, glial fibrillary acidic protein and total tau protein. We assessed whether these markers were associated with the severity of the acute COVID-19 illness and with post-acute neuropsychiatric symptoms (as measured by the Patient Health Questionnaire for depression, the General Anxiety Disorder assessment for anxiety, the Montreal Cognitive Assessment for objective cognitive deficit and the cognitive items of the Patient Symptom Questionnaire for subjective cognitive deficit) at 6 months and 1 year post-hospital discharge from COVID-19. No robust associations were found between markers of nervous system injury and severity of acute COVID-19 (except for an association of small effect size between duration of admission and neurofilament light) nor with post-acute neuropsychiatric symptoms. These results suggest that ongoing neuropsychiatric symptoms are not due to ongoing neural injury.

## Teaser

COVID-19 is associated with raised neural injury markers and neuropsychiatric sequelae. It is unknown whether post-acute neural injury is linked to neuropsychiatric symptoms. Taquet *et al*. showed that there was no robust link between the two, suggesting that neuropsychiatric symptoms of post-acute COVID illness are not caused by ongoing neural injury.

## Graphical Abstract

**Graphical Abstract:**
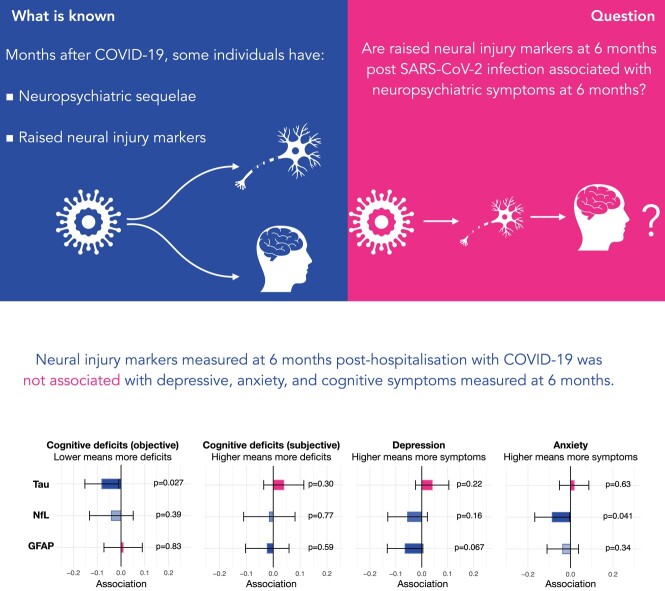

## Introduction

In the weeks and months after severe acute respiratory syndrome coronavirus 2 (SARS-CoV-2) infection, a proportion of patients experience neuropsychiatric symptoms including depression, anxiety and cognitive deficits (also known as ‘brain fog’).^1-6^ The risk is particularly marked in patients hospitalized at the time of their COVID-19,^1,7^ and an increased number of new cases are still being diagnosed months after the acute infection.^3^

While the exact mechanisms underpinning such associations remain largely unknown,^8^ recent studies have started to shed light on how COVID-19 might lead to neuropsychiatric sequelae. An animal study has found that microvascular brain pathology following COVID-19 can be caused by SARS-CoV-2 main protease cleaving nuclear factor-κB essential modulator, thus inducing the death of brain endothelial cells.^9^ Another study in animals found that mild SARS-CoV-2 infection was associated with elevated CSF levels of the cytokine CCL11, which can cause hippocampal microglial activation and impaired neurogenesis, as well as demyelination.^10^ An autopsy study of the brain of individuals infected with SARS-CoV-2 found multi-focal vascular damage accompanied by endothelial cell activation and evidence of neuroinflammation.^11^ These studies raise the possibility that the associations between SARS-CoV-2 infection and neuropsychiatric sequelae are mediated by neuronal injury (as a direct result of neuroinflammation and/or due to ischaemic injury).

Several studies have shown that nervous system injury markers [including neurofilament light (NfL) and glial fibrillary acidic protein (GFAP)] are raised after COVID-19.^12-19^ In particular, a recent study showed that both NfL and tau remain elevated 4 months after admission to hospital for COVID-19 in some patients.^19^ This raises the possibility of ongoing neural injury in the post-acute phase of the illness and might explain why the risk of some neuropsychiatric symptoms (notably cognitive deficits) continues to be raised up to 2 years after a COVID-19 diagnosis.^3^ However, the association between neural injury markers and neuropsychiatric outcomes following COVID-19 is unclear. One study of 61 patients post-COVID reported that NfL and GFAP are raised in patients with post-COVID neurological symptoms.^18^ However, another study of 121 patients with COVID-19 found that early GFAP levels were correlated with post-acute neurological symptoms but found no association with NfL.^20^ Yet, a third study based on 97 participants found no association between GFAP and NfL (measured both acutely and 7 months post-infection) and self-reported cognitive deficit.^21^ The relatively small sample size of these studies precluded adjustment for important confounding factors including physical and psychiatric comorbidities, as well as history of neurological and neurovascular disease. The latter is particularly important as raised levels of post-COVID neural injury markers might reflect pre-existing neurovascular impairment, which is a known risk factor for more severe COVID-19 illness.^22^

In this study, we investigated whether post-acute nervous system injury markers are associated with depressive symptoms [measured by the Patient Health Questionnaire (PHQ-9)], anxiety [measured by the General Anxiety Disorder (GAD-7) assessment] and cognitive deficits [measured by the Montreal Cognitive Assessment (MoCA) and the cognitive items of the Patient Symptom Questionnaire (C-PSQ)]. We also investigated whether the severity of acute COVID-19 illness is associated with raised levels of nervous system injury markers.

## Materials and methods

### Data

We used data from the Post-hospitalization COVID-19 (PHOSP-COVID, ISCTN Registry ISRCTN10980107) study, which is a large-scale prospective cohort study of adults (aged ≥18 years) discharged from a hospital in the UK with a clinical diagnosis of COVID-19 (this analysis included participants discharged between 29 January 2020 and 20 November 2021).^23^ The study, which involved data collection at baseline (i.e. during hospitalization), at 2–7 months after discharge (which we refer to as the 6-month follow-up for simplicity) and 12 months after hospital discharge, consisted of an extensive assessment of patients’ clinical data, with collected measurements including routine clinical data, results of blood tests and clinical questionnaires.

For each participant, data from their hospital admission were recorded. These included the following variables, which we used as COVID-19 severity markers: World Health Organization (WHO) clinical progression scale (based on the level of oxygenation assistance required),^24^ National Early Warning Scores (NEWS) representing the degree of departure from the normal range of physical observations, duration of hospital admission, diagnosis of pulmonary embolism during hospitalization, admission to intensive care, presence of altered consciousness or confusion during admission and recovery clusters identified in a previous study based on the PHOSP cohort.^2^

Clinical scales representing neuropsychiatric health were measured for each participant at the 6-month follow-up and for a subset of participants at the 12-month follow-up. These included the PHQ-9, GAD-7, MoCA and C-PSQ (see Supplementary material for details).

### Markers of nervous system injury

At the 6-month follow-up, a subset of PHOSP-COVID participants had plasma investigated for levels of peripheral markers of nervous system injury (tau protein and NfL) and astrocytic activation (GFAP). Samples were stored at −80° at study sites before being transported on dry ice and analysed at the UK Dementia Research Institute Biomarker Factory at University College London, UK, using the multiplexed single molecule array (Simoa) Human Neurology 4-Plex B assay (Quanterix, Billerica, MA, USA). All samples were analysed in one round of experiments with one batch of reagents. Intra-assay coefficients of variation of tau, NfL and GFAP were below 10% (6.22% for tau, 4.83% for NfL and 7.17% for GFAP). Limits of detection were 0.0371 pg/ml for tau, 0.0962 pg/ml for NfL and 1.18 pg/ml for GFAP. Two tau samples and two GFAP samples failed this criterion and were not included in the study. The lower limit of quantification was 0.125 pg/ml for tau, 0.500 pg/ml for NfL and 9.38 pg/ml for GFAP. All samples met this criterion. The four-plex panel also measures UCH-L1, but this particular marker did not pass quality control criteria, with coefficients of variation exceeding 20%.

We also tested the quality of the nervous system injury marker data by, firstly, reproducing their known association with age (positive correlation for NfL and GFAP^15^). We did this by calculating the correlation between age on measurement and the log of the values of nervous system injury markers and by visual inspection. Secondly, a subset of subjects had their markers of nervous system injury measured twice on the same day using separate blood samples. This allowed us to estimate the test–retest reliability of each marker of nervous system injury.

### Statistical analyses

We assessed both the association between markers of nervous system injury and post-acute neuropsychiatric outcomes (Objective 1) and the association between markers of COVID-19 severity and markers of nervous system injury (Objective 2). Both were assessed using separate sets of standard linear regressions, adjusted for a range of possible confounders, including demographics (sex, age and ethnicity) and each of a number of comorbidities (diagnoses of respiratory, rheumatological, cardiovascular and gastrointestinal condition, history of a cerebrovascular accident, dementia, Parkinson’s disease, depression, anxiety, chronic fatigue syndrome, chronic pain, diabetes, hypothyroidism/hyperthyroidism or other chronic metabolic/endocrine disorders, chronic kidney disease, cancer and history of chronic infectious diseases). In addition, the association between markers of nervous system injury and COVID-19 neuropsychiatric outcomes was adjusted for factors thought to be associated with clinical outcomes: educational level, household income, marital status and whether English was a patient’s first language.

In all analyses, both the dependent and the independent variables were standardized, so that the coefficients reflected how many standard deviations (SDs) the dependent variable would change if the independent variable changed by 1 SD. The markers of nervous system injury were scaled to a logarithmic scale as their distribution was exponential-like. Recovery clusters were encoded as a categorical variable, and their effects were reported against the cluster associated with mild mental and physical impairment. Statistical significance was set to two-sided *P*-values <0.05. Bonferroni correction for multiple comparisons was applied (accounting for eight comparisons for Objective 1 and three comparisons for Objective 2). Uncorrected *P*-values were also reported. Analyses were performed in R version 4.2.0.

### Secondary analyses

For each study objective, we performed the following two robustness analyses:

1. Using a single model incorporating all independent variables (i.e. all markers of nervous system injury for Objective 1 and all markers of COVID-19 severity for Objective 2), rather than running separate regression for each independent variable as in the primary analysis.
2. Imputing missing data for all variables (dependent, independent and confounders). This was achieved using a multiple imputation model with 20 chains and 5 iterations.^25^

In addition, the associations between neural injury markers and different cognitive domains measured by the MoCA at 6 months were assessed in the same way as for the association with the total MoCA score.

Details about the robustness analyses can be found in the Supplementary material.

## Results

We identified a total of 891 patients from the PHOSP-COVID cohort who had measurements of all 3 markers of neural injury studied at the 6-month study visit {median [interquartile range (IQR)] follow-up: 187 [150–214] days} post-discharge from hospital [mean (SD) age at hospital admission 57.3 (12.5) years old, 35.0% female], of which 507 individuals also had a follow-up at 12 months [mean (SD) age at hospital admission 58.8 (12.0) years old, 33.5% female]. Table 1 summarizes the baseline characteristics of the cohort (see also Supplementary Tables 1 and 2 for all baseline characteristics as well as those of the cohort with a 12-month follow-up). Table 2 summarizes the markers of COVID-19 severity, neuropsychiatric outcomes and the values of neural injury markers.

**Table 1:** Baseline characteristics for the cohort used in this study and for all other PHOSP-COVID participants

Cohort	PHOSP-COVID participants with neural injury markers	All other PHOSP-COVID participants
Cohort size	891	1806
Sociodemographic factors		
Age, mean (SD)	57.3 (12.5)	58.2 (12.8)
Sex, *n* (%)		
Female	312 (35.0)	637 (35.3)
Male	535 (60.0)	958 (53.0)
Unknown	44 (4.9)	211 (11.7)
Race, *n* (%)		
White	668 (75.0)	1199 (66.4)
Asian	122 (13.7)	188 (10.4)
Black	47 (5.3)	130 (7.2)
Mixed	14 (1.6)	37 (2.0)
Other	24 (2.7)	75 (4.2)
Unknown	16 (1.8)	177 (9.8)
Comorbidities, *n* (%)		
Cancer	61 (6.8)	122 (6.8)
Cardiovascular condition	372 (41.8)	791 (43.8)
Myalgic encephalomyelitis/chronic fatigue syndrome (ME/CFS)/fibromyalgia/chronic pain	40 (4.5)	91 (5.0)
Chronic kidney disease	33 (3.7)	73 (4.0)
Cerebrovascular accident	33 (3.7)	70 (3.9)
Dementia	<10	<10
Diabetes	166 (18.6)	351 (19.4)
Metabolic/endocrine disorder	65 (7.3)	152 (8.4)
Gastrointestinal condition	179 (20.1)	371 (20.5)
Infectious disease	21 (2.4)	49 (2.7)
Parkinson’s disease	<10	<10
Depression or anxiety	151 (16.9)	308 (17.1)
Respiratory condition	254 (28.5)	457 (25.3)
Rheumatological condition	121 (13.6)	273 (15.1)

Only a subset of characteristics is shown. For all other characteristics (including educational level, household income, marital status and whether English is the participant’s first language), please see Supplementary Table 1.

**Table 2:** Markers of COVID-19 severity and neuropsychiatric outcomes for the cohort used in this study and for all other PHOSP-COVID participants

Cohort	PHOSP-COVID participants with neural injury markers	All other PHOSP-COVID participants
COVID-19 severity markers		
WHO, mean (SD)	2.45 (1.07)	2.42 (0.915)
NEWS, mean (SD)	3.71 (2.35)	3.78 (2.29)
Duration of hospital admission, mean (SD)	14.8 (19.2)	14.3 (21.6)
Pulmonary embolism, *n* (%)	83 (9.3)	154 (8.5)
Altered consciousness/confusion, *n* (%)	72 (8.1)	206 (11.4)
ICU admission, *n* (%)	304 (34.1)	471 (26.1)
COVID-19 recovery clusters, *n* (%)		
Mild impairment	123 (13.8)	207 (11.5)
Moderate and cognitive impairment	195 (21.9)	318 (17.6)
Severe impairment	76 (8.5)	108 (6.0)
Very severe impairment	288 (32.3)	376 (20.8)
Unknown	209 (23.5)	797 (44.1)
Clinical scales at 6 months, mean (SD)		
MoCA	25.6 (3.68)	25.6 (3.47)
GAD-7	5.32 (5.96)	5.38 (5.57)
PHQ-9	6.99 (6.57)	7.09 (6.56)
C-PSQ	2.05 (2.03)	2.09 (2.07)
Neural injury markers, mean (SD)		
GFAP	4.28 (0.91)	N/A
Tau protein	1.70 (0.78)	N/A
NfL	2.25 (0.72)	N/A
Follow-up		
Number at 6-month follow-up, *n* (%)	891 (100)	1573 (87.1)
Time at 6 months, median (IQR), days	187 (150–214)	162 (123–200)
Number at 12-month follow-up, *n* (%)	507 (56.9)	442 (24.5)
Time at 12 months, median (IQR), days	401 (375–424)	397 (365–426)

For the cohort used in this study, the value of neural injury markers is also reported.

Measurements of neural injury were found to be reliable both in terms of their test–retest reliability (test–retest correlations of 0.98 for NfL, 0.74 for tau and 0.99 for GFAP, *P* < 0.001 for all three) and in terms of their association with age (correlation with age of 0.57 for NfL and 0.38 for GFAP, *P* < 0.001 for both; see Supplementary Fig. 1).

**Figure 1:**
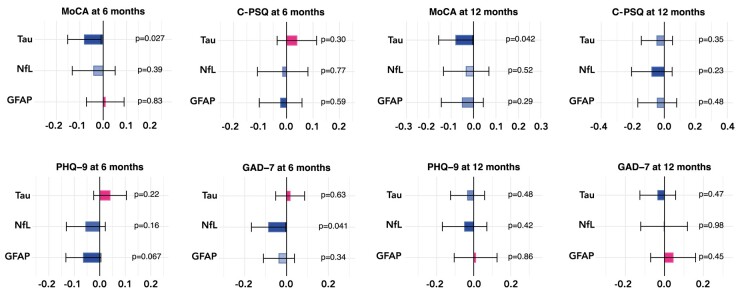
**Associations between markers of nervous system injury and post-acute neuropsychiatric features measured at 6 and 12 months**. Each bar represents the number of SDs by which the neuropsychiatric scale varies when the level of the nervous system injury marker varies by 1 SD. The error bars indicate 95% CIs. Uncorrected *P*-values are reported next to each bar, and Bonferroni-corrected *P*-values meeting statistical significance thresholds are flagged as follows: *<0.05, **<0.01 and ***<0.001. See Supplementary Figs 2 and 3 for the same results based on different statistical models.

We found no evidence that post-acute nervous system injury biomarkers associate with cognitive and neuropsychiatric symptoms in the post-acute phase of severe COVID illness after correcting for multiple comparisons (see Fig. 1 and Supplementary Tables 3 and 4). Uncorrected *P*-values <0.05 were observed for a negative association between tau and the MoCA at 6 [beta = −0.080, 95% confidence interval (CI) −0.0091 to −0.15, *P* = 0.027] and 12 months (beta = −0.080, 95% CI −0.0031 to −0.16, *P* = 0.042). These results were robust when all markers of nervous system injury were used as independent variables within a single regression model (Supplementary Fig. 2) and when missing data were imputed (Supplementary Fig. 3). No significant associations were found for any of the subdomains of the MoCA (Supplementary Table 5).

**Figure 2:**
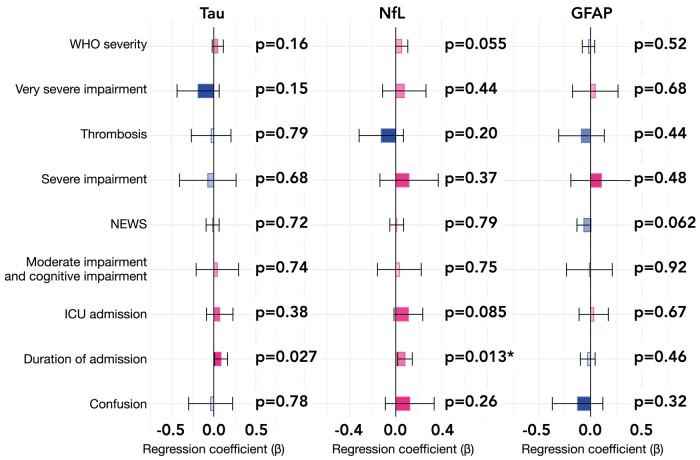
**Associations between markers of nervous system injury and markers of severity of the acute COVID-19 illness**. Each bar represents the number of SDs by which the levels of nervous system injury marker vary when the marker of severity is present versus absent (for dichotomous variables, i.e. the presence of thrombosis, ICU admission and confusion) or when the marker of severity varies by 1 SD (for non-dichotomous variables, i.e. WHO class, NEWS score and the duration of admission). The error bars indicate 95% CIs. Uncorrected *P*-values are reported next to each bar, and Bonferroni-corrected *P*-values meeting statistical significance thresholds are flagged as follows: *<0.05, **<0.01 and ***<0.001. WHO severity refers to the World Health Organization classification of COVID-19 severity (in terms of oxygen requirement). Very severe impairment, severe impairment and moderate impairment with cognitive impairment refer to predefined clusters of post-COVID impairment.^2^ See Supplementary Figs 4 and 5 for the same results based on different statistical models.

We found that in the models linking COVID-19 severity (including severity of ongoing impairment as encoded by recovery clusters) to markers of nervous system injury (Objective 2), the only significant association was between the duration of admission and NfL (beta = 0.080, 95% CI 0.017–0.14, *P* = 0.013, Bonferroni-adjusted *P* = 0.039; see Fig. 2). The same relationship existed for tau although the latter did not survive correction for multiple comparisons (beta = 0.087, 95% CI 0.010–0.16, *P* = 0.027, Bonferroni-corrected *P* = 0.081). The association between duration of admission and NfL was small in effect size: a difference in duration of hospitalization of 1 SD (i.e. 20.7 days) was associated with a difference in NfL of 0.080 SD in the log-domain (which is of similar magnitude as the difference in NfL observed between two individuals with an age difference of only 16.6 days). These results were robust when all markers of severity were used as independent variables within a single regression model (Supplementary Fig. 4) and when missing data were imputed (Supplementary Fig. 5).

## Discussion

In this prospective cohort study of COVID-19 survivors, we found that markers of nervous system injury in plasma measured 6 months after hospital admission were not associated with post-acute neuropsychiatric features (cognitive deficits, depressive and anxiety symptoms) and not robustly associated with severity of the acute illness.

If post-acute neuropsychiatric consequences of COVID-19 are not explained by ongoing brain injury, then other mechanisms are needed to explain why new cases of neuropsychiatric sequelae (especially cognitive deficits) are being diagnosed up to 2 years after infection.^3^ A first possibility is that these sequelae are the product of acute immune or vascular events in the CNS, which lead to persistent symptoms.^26^ The ongoing diagnosis of new cases in the post-acute phase of the illness would then reflect delayed presentation of persistent symptoms that originated in the acute phase. A similar scenario has been observed in survivors of out-of-hospital cardiac arrest in whom evidence of acute brain injury predicted poor neurological outcomes 6 months later.^27^ This explanation would also be consistent with the observation that self-reported post-acute neurological symptoms are associated with higher GFAP levels measured in the acute phase but not with those measured in the post-acute phase.^20^ A second possibility is that post-acute neuropsychiatric sequelae are the results of ongoing neuropathological processes that are not captured by markers of nervous system injury, such as impaired hippocampal neurogenesis.^28,29^ A third possibility is that some post-acute neuropsychiatric sequelae of COVID-19 are functional rather than structural in aetiology.^30^ Such functional neuropsychiatric sequelae can be precipitated by acute psychosocial stressors in the context of COVID-19.^30^ A wider range of investigations is needed in individual patients to make such a diagnosis, and if confirmed, targeted therapy should be offered to those individuals. These three possible explanations are not mutually exclusive, and different neuropsychiatric presentations might be underpinned by different mechanisms in different people. In particular, the different risk trajectories observed for anxiety and mood disorders on the one hand, and cognitive deficits on the other hand, suggest that different mechanisms might be at play.^3^

The absence of robust associations between severity of the acute COVID-19 illness and post-acute markers of nervous system injury might suggest that no ongoing nervous system injury occurs 6 months after COVID-19. The lack of healthy controls in this study (as the primary focus was to compare COVID-19 patients with versus without neuropsychiatric sequelae) prevents us from ruling this possibility out. However, it would not be compatible with the raised NfL in the post-acute phase of moderate-to-severe COVID-19 observed in another study.^19^ There are two reasons why NfL may remain elevated in the absence of neuropsychiatric symptoms. First, it might reflect an ongoing degree of brain injury that is too mild to have clinical manifestations. The lack of a standardized approach to measure NfL means that it is impossible to appreciate the clinical significance of a specific level reported in a study. Second, raised NfL might reflect ongoing injury in areas of the nervous system not involved in cognition. In particular, injury might be occurring in the peripheral nervous system, which would help explain the association between COVID-19 and peripheral nerve disorders and myopathy.^7^ Consistent with this possibility are the observations, in COVID-19 patients, of a raised NfL level associated with critical illness polyneuropathy/myopathy^31^ (for which duration of admission is an established risk factor^32^) and of a raised serum NfL level in the absence of raised levels of NfL in theCSF.^17^

This study has several strengths, including the largest sample size to date of measurements of nervous system injury markers in patients with COVID-19, the assessment of both objective and subjective cognitive deficits and the adjustment for a wide range of confounders. However, it also has limitations besides the lack of healthy controls and the absence of measurements of nervous system injury markers in the acute phase, as already discussed. The main limitation (present in all studies of the association between COVID-19 and markers of nervous system injury to date) is that no pre-COVID measurements of nervous system injury are available, so that reverse causation might be present. Second, only peripheral markers of nervous system injury were measured, and large-scale studies with CSF measurements are needed to better delineate the location of injury captured by those markers. Third, the neuropsychiatric features were limited to those measured by four instruments: PHQ-9, GAD-7, MoCA and C-PSQ. While the first two are validated for depression and anxiety disorders, the MoCA is validated only in the context of dementia. This implies that post-acute neural injury markers might be associated with other neuropsychiatric features not captured by those instruments such as cognitive deficits in domains or to a degree not adequately measured by the MoCA and C-PSQ, psychotic features, sleep problems and fatigue. Studies based on more comprehensive neuropsychological assessments are therefore needed. Lastly, in this study, we were only able to report on individuals with severe COVID illness who required hospitalization in the UK. However, individuals who remained ambulatory are also known to report significant long-term neuropsychiatric symptoms.^33^ Thus, a future research direction is to explore the evidence for neural injury in this substantial group of patients.

In summary, our results provide evidence from the largest sample size to date that post-acute neuropsychiatric symptoms (in terms of depression, anxiety and cognitive deficits measured by PHQ-9, GAD-7, MoCA and C-PSQ) are not the product of ongoing neural injury. The significant burden of these conditions on COVID-19 survivors necessitates further research into its causes.

## Supplementary Material

fcad357_Supplementary_Data

## Data Availability

The PHOSP study data are available for independent analysis by researchers. The protocol, consent form, definition and derivation of clinical characteristics and outcomes, training materials, regulatory documents, information about requests for data access and other relevant study materials are available at https://www.phosp.org/.
