# Supplementary material for: Post-acute COVID-19 neuropsychiatric symptoms are not associated with ongoing nervous system injury
Source: Brain Commun. 2023 Dec 27;6(1):fcad357. doi: 10.1093/braincomms/fcad357 (PMC10789589; doi:10.1093/braincomms/fcad357)
Supplement: fcad357_Supplementary_Data [file fcad357_supplementary_data.pdf]

Supplementary material

Post-acute COVID-19 neuropsychiatric symptoms are not associated with ongoing nervous system injury

PHOSP-COVID Collaborative Group

Core Management Group

Chief Investigator C E Brightling, *Members* R A Evans (Lead Co-I), L V Wain (Lead Co-I), J D Chalmers, V C Harris, L P Ho, A Horsley, M Marks, K Poinasamy, B Raman, A Shikotra, A Singapuri

PHOSP-COVID Study Central Coordinating Team

C E Brightling (Chief Investigator), R A Evans (*Lead Co-I*), L V Wain (*Lead Co-I*), R Dowling, C Edwardson, O Elneima, S Finney, N J Greening, B Hargadon, V Harris, L Houchen--Wolloff, O C Leavy, H J C McAuley, C Overton, T Plekhanova, R M Saunders, M Sereno, A Singapuri, A Shikotra, C Taylor, S Terry, C Tong, B Zhao

Steering Committee

Co-chairs D Lomas, E Sapey*, Institution representatives* C Berry, C E Bolton, N Brunskill, E R Chilvers, R Djukanovic, Y Ellis, D Forton, N French, J George, N A Hanley, N Hart, L McGarvey, N Maskell, H McShane, M Parkes, D Peckham, P Pfeffer, A Sayer, A Sheikh, A A R Thompson, N Williams and core management group representation

Executive Board

Chair C E Brightling, representation from the core management group, each working group and platforms

Platforms

Bioresource

W Greenhalf (*Co-Lead*), M G Semple (*Co-Lead*), M Ashworth, H E Hardwick, L Lavelle-Langham, W Reynolds, M Sereno, R M Saunders, A Singapuri, V Shaw, A Shikotra, B Venson, L V Wain

Data Hub

A B Docherty (*Co-Lead*), E M Harrison (*Co-Lead*), A Sheikh (*Co-Lead*), J K Baillie, C E Brightling, L Daines, R Free, R A Evans, S Kerr, O C Leavy, N I Lone, H J C McAuley, R Pius, J Quint, M Richardson, M Sereno, M Thorpe, L V Wain

Imaging Alliance

M Halling-Brown (*Co-Lead*), F Gleeson (*Co-Lead*), J Jacob (*Co-Lead*), S Neubauer (*Co-Lead*) B Raman (*Co-Lead*) S Siddiqui (*Co-Lead*) J M Wild (*Co-Lead*), S Aslani, P Jezzard, H Lamlum, W Lilaonitkul, E Tunnicliffe, J Willoughby

Omics

L V Wain (*Co-Lead)*, J K Baillie (*Co-Lead*), H Baxendale, C E Brightling, M Brown, J D Chalmers, R A Evans, B Gooptu, W Greenhalf, H E Hardwick, R G Jenkins, D Jones, I Koychev, C Langenberg, A Lawrie, P L Molyneaux, A Shikotra, J Pearl, M Ralser, N Sattar, R M Saunders, J T Scott, T Shaw, D Thomas, D Wilkinson

Working Groups

Airways

L G Heaney (*Co-Lead*), A De Soyza (*Co-Lead*), D Adeloye, C E Brightling, J S Brown, J Busby, J D Chalmers, C Echevarria, L Daines, O Elneima, RA Evans, J Hurst, P Novotny, P Pfeffer, K Poinasamy, J Quint, I Rudan, E Sapey, M Shankar-Hari, A Sheikh, S Siddiqui, S Walker, B Zheng

Brain

J R Geddes (*Lead*), M Hotopf *(Co-Lead),* K Abel, R Ahmed, L Allan, C Armour, D Baguley, D Baldwin, C Ballard, K Bhui, G Breen, M Broome, T Brugha, E Bullmore, D Burn, F Callard, J Cavanagh, T Chalder, D Clark, A David, B Deakin, H Dobson, B Elliott, J Evans, R Francis, E Guthrie, P Harrison, M Henderson, A Hosseini, N Huneke, M Husain, T Jackson, I Jones, T Kabir, P Kitterick, A Korszun, I Koychev, J Kwan, A Lingford-Hughes, P Mansoori, H McAllister-Williams, K McIvor, L Milligan, R Morriss, E Mukaetova-Ladinska, K Munro, A Nevado-Holgado, T Nicholson, S Paddick, C Pariante, J Pimm, K Saunders, M Sharpe, G Simons, R Upthegrove, S Wessely

Cardiac

G P McCann (*Lead*), S Amoils, C Antoniades, A Banerjee, R Bell, A Bularga, C Berry, P Chowienczyk, J P Greenwood, A D Hughes, K Khunti, L Kingham, C Lawson, K Mangion, N L Mills, A J Moss, S Neubauer, B Raman, A N Sattar, C L Sudlow, M Toshner,

Immunology

P J M Openshaw (*Lead*), D Altmann, J K Baillie, R Batterham, H Baxendale, N Bishop, C E Brightling, P C Calder, R A Evans, J L Heeney, T Hussell, P Klenerman, J M Lord, P Moss, S L Rowland-Jones, W Schwaeble, M G Semple, R S Thwaites, L Turtle, L V Wain, S Walmsley, D Wraith

Intensive Care

M J Rowland (*Lead*), A Rostron (*Co-Lead*), J K Baillie, B Connolly, A B Docherty, N I Lone, D F McAuley, D Parekh, A Rostron, J Simpson, C Summers

Lung Fibrosis

R G Jenkins (*Co-Lead*), J Porter (*Co-Lead*), R J Allen, R Aul, J K Baillie, S Barratt, P Beirne, J Blaikley, R C Chambers, N Chaudhuri, C Coleman, E Denneny, L Fabbri, P M George, M Gibbons, F Gleeson, B Gooptu, B Guillen Guio, I Hall, N A Hanley, L P Ho, E Hufton, J Jacob, I Jarrold, G Jenkins, S Johnson, M G Jones, S Jones, F Khan, P Mehta, J Mitchell, P L Molyneaux, J E Pearl, K Piper Hanley, K Poinasamy, J Quint, D Parekh,

P Rivera-Ortega, L C Saunders, M G Semple, J Simpson, D Smith, M Spears, L G Spencer, S Stanel, I Stewart, A A R Thompson, D Thickett, R Thwaites, L V Wain, S Walker, S Walsh, J M Wild, D G Wootton, L Wright

Metabolic

S Heller (*Co-Lead*), M J Davies (*Co-Lead*), H Atkins, S Bain, J Dennis, K Ismail, D Johnston, P Kar, K Khunti, C Langenberg, P McArdle, A McGovern, T Peto, J Petrie, E Robertson, N Sattar, K Shah, J Valabhji, B Young

Pulmonary and Systematic Vasculature

L S Howard (*Co-Lead*), Mark Toshner (*Co-Lead*), C Berry, P Chowienczyk, D Lasserson, A Lawrie, O C Leavy, J Mitchell, L Price, J Quint, J Rossdale, N Sattar, C Sudlow, A A R Thompson, J M Wild, M Wilkins

Rehabilitation, Sarcopenia and Fatigue

S J Singh (*Co-Lead*), W D-C Man (*Co-Lead*), J M Lord (*Co-Lead*), N J Greening (*Co-Lead*), T Chalder (*Co-Lead*), J T Scott (*Co-Lead*), N Armstrong, E Baldry, M Baldwin, N Basu, M Beadsworth, L Bishop, C E Bolton, A Briggs, M Buch, G Carson, J Cavanagh, H Chinoy, E Daynes, S Defres, R A Evans, P Greenhaff, S Greenwood, M Harvie, M Husain, S MacDonald, A McArdle, H J C McAuley, A McMahon, M McNarry, C Nolan, K O'Donnell, D Parekh, Pimm, J Sargent, L Sigfrid, M Steiner, D Stensel, A L Tan, J Whitney, D Wilkinson, D Wilson, M Witham, D G Wootton, T Yates

Renal

D Thomas (*Lead*), N Brunskill (*Co-Lead*), S Francis (*Co-Lead*), S Greenwood (*Co-Lead*), C Laing (*Co-Lead*), K Bramham, P Chowdhury, A Frankel, L Lightstone, S McAdoo, K McCafferty, M Ostermann, N Selby, C Sharpe, M Willicombe

Local Clinical Centre PHOSP-COVID trial staff

(listed in alphabetical order)

Airedale NHS Foundation Trust

A Shaw (PI), L Armstrong, B Hairsine, H Henson, C Kurasz, L Shenton

Aneurin Bevan University Health Board

S Fairbairn (PI), A Dell, N Hawkings, J Haworth, M Hoare, A Lucey, V Lewis, G Mallison, H Nassa, C Pennington, A Price, C Price, A Storrie, G Willis, S Young

Barts Health NHS Trust & Queen Mary University of London

P Pfeffer (PI), K Chong-James, C David, W Y James, A Martineau, O Zongo

Barnsley Hospital NHS Foundation Trust

A Sanderson (PI)

Belfast Health and Social Care Trust & Queen's University Belfast

L G Heaney (PI), C Armour, V Brown, T Craig, S Drain, B King, N Magee, D McAulay, E Major, L McGarvey, J McGinness, R Stone

Betsi Cadwaladr University Health Board

A Haggar (PI), A Bolger, F Davies, J Lewis, A Lloyd, R Manley, E McIvor, D Menzies, K Roberts, W Saxon, D Southern, C Subbe, V Whitehead

Borders General Hospital, NHS Borders

H El-Taweel (PI), J Dawson, L Robinson

Bradford Teaching Hospitals NHS Foundation Trust

D Saralaya (PI), L Brear, K Regan, K Storton

Cambridge University Hospitals NHS Foundation Trust, NIHR Cambridge Clinical Research Facility & University of Cambridge

J Fuld (PI), A Bermperi, I Cruz, K Dempsey, A Elmer, H Jones, S Jose, S Marciniak, M Parkes, C Ribeiro, J Taylor, M Toshner, L Watson, J Worsley

Cardiff and Vale University Health Board

R Sabit (PI), L Broad, A Buttress, T Evans, M Haynes, L Jones, L Knibbs, A McQueen, C Oliver, K Paradowski, J Williams

Chesterfield Royal Hospital NHS Trust

E Harris (PI), C Sampson

Cwm Taf Morgannwg University Health Board

C Lynch (PI), E Davies, C Evenden , A Hancock, K Hancock, M Rees , L Roche, N Stroud, T Thomas-Woods

East Cheshire NHS Trust

M Babores (PI), J Bradley-Potts, M Holland, N Keenan, S Shashaa, H Wassall

East Kent Hospitals University NHS Foundation Trust

E Beranova (PI), H Weston (PI), T Cosier, L Austin, J Deery, T Hazelton, C Price, H Ramos, R Solly, S Turney

Gateshead NHS Trust

L Pearce (PI), W McCormack, S Pugmire, W Stoker, A Wilson

Guy’s and St Thomas’ NHS Foundation Trust

N Hart (PI), LA Aguilar Jimenez, G Arbane, S Betts, K Bisnauthsing, A Dewar, P Chowdhury, A Dewar, G Kaltsakas, H Kerslake, MM Magtoto, P Marino, LM Martinez, M Ostermann, J Rossdale, TS Solano, E Wynn

Hampshire Hospitals NHS Foundation Trust

N Williams (PI), W Storrar (PI), M Alvarez Corral, A Arias, E Bevan, D Griffin, J Martin, J Owen,

S Payne, A Prabhu, A Reed, C Wrey Brown

Harrogate and District NHD Foundation Trust

C Lawson (PI), T Burdett, J Featherstone, A Layton, C Mills, L Stephenson,

Hull University Teaching Hospitals NHS Trust & University of Hull

N Easom (PI), P Atkin, K Brindle, M G Crooks, K Drury, R Flockton, L Holdsworth, A Richards, D L Sykes, S Thackray-Nocera, C Wright

Hywel Dda University Health Board

K E Lewis (PI), A Mohamed (PI), G Ross (PI), S Coetzee, K Davies, R Hughes, R Loosley, L O’Brien, Z Omar, H McGuinness, E Perkins, J Phipps, A Taylor, H Tench, R Wolf-Roberts

Imperial College Healthcare NHS Trust & Imperial College London

L S Howard (PI), O Kon (PI), D C Thomas (PI), S Anifowose, L Burden, E Calvelo, B Card, C Carr, E R Chilvers, D Copeland, P Cullinan, P Daly, L Evison, T Fayzan, H Gordon, S Haq, R G Jenkins, C King, K March, M Mariveles, L McLeavey, N Mohamed, S Moriera, U Munawar, J Nunag, U Nwanguma, L Orriss- Dib, A Ross, M Roy, E Russell, K Samuel, J Schronce, N Simpson, L Tarusan, C Wood, N Yasmin

Kettering General Hospital NHS Trust

R Reddy (PI), A-M, Guerdette, M Hewitt, K Warwick, S White

King’s College Hospital NHS Foundation Trust & Kings College London

A M Shah (PI), C J Jolley (PI), O Adeyemi, R Adrego, H Assefa-Kebede, J Breeze, M Brown, S Byrne, T Chalder, P Dulawan, N Hart, A Hayday, A Hoare, A Knighton, M Malim, S Patale, I Peralta, N Powell, A Ramos, K Shevket, F Speranza, A Te

Leeds Teaching Hospitals & University of Leeds

P Beirne (PI), A Ashworth, J Clarke, C Coupland, M Dalton, E Wade, C Favager, J Greenwood, J Glossop, L Hall, T Hardy, A Humphries, J Murira, D Peckham, S Plein, J Rangeley, G Saalmink, A L Tan, B Whittam, N Window, J Woods,

Lewisham & Greenwich NHS Trust

G Coakley (PI)

Liverpool University Hospitals NHS Foundation Trust & University of Liverpool

D G Wootton (PI), L Turtle (PI), L Allerton, AM All, M Beadsworth, A Berridge, J Brown, S Cooper, A Cross, S Defres, S L Dobson, J Earley, N French, W Greenhalf, H E Hardwick, K Hainey, J Hawkes, V Highett, S Kaprowska, AL Key, L Lavelle-Langham, N Lewis-Burke, G Madzamba, F Malein, S Marsh, C Mears, L Melling, M J Noonan, L Poll, J Pratt, E Richardson, A Rowe, M G Semple, V Shaw, K A Tripp, L O Wajero, S A Williams-Howard, J Wyles,

London North West University Healthcare NHS Trust

S N Diwanji (PI), P Papineni (PI), S Gurram, S Quaid, G F Tiongson, E Watson

Manchester University NHS Foundation Trust & University of Manchester

B Al-Sheklly (PI), A Horsley (PI), C Avram, J Blaikely, M Buch, N Choudhury, D Faluyi, T Felton, T Gorsuch, N A Hanley, T Hussell, Z Kausar, N Odell, R Osbourne, K Piper Hanley, K Radhakrishnan, S Stockdale

Newcastle upon Tyne Hospitals NHS Foundation Trust & University of Newcastle

A De Soyza (PI), C Echevarria (PI), A Ayoub, J Brown, G Burns, G Davies, H Fisher, C Francis, A Greenhalgh, P Hogarth, J Hughes, K Jiwa, G Jones, G MacGowan, D Price, A Sayer, J Simpson, H Tedd, S Thomas, S West, M Witham, S Wright, A Young

NHS Dumfries and Galloway

M J McMahon (PI), P Neill

NHS Greater Glasgow and Clyde Health Board & University of Glasgow

D Anderson (PI), H Bayes (PI), C Berry (PI), D Grieve (PI), I B McInnes (PI), N Basu, A Brown, A Dougherty, K Fallon, L Gilmour, K Mangion, A Morrow, K Scott, R Sykes

NHS Highland

E K Sage (PI), F Barrett, A Donaldson

NHS Lanarkshire

M Patel (PI), D Bell, A Brown, M Brown, R Hamil, K Leitch, L Macliver, J Quigley, A Smith, B Welsh

NHS Lothian & University of Edinburgh

G Choudhury (PI), J K Baillie, S Clohisey, A Deans, A B Docherty, J Furniss, E M Harrison, S Kelly, N I Lone, A Sheikh

NHS Tayside & University of Dundee

J D Chalmers (PI), D Connell, A Elliott, C Deas, J George, S Mohammed, J Rowland, A R Solstice, D Sutherland, C J Tee

North Bristol NHS Trust & University of Bristol

N Maskell (PI), D Arnold, S Barrett, H Adamali, A Dipper, S Dunn, A Morley, L Morrison, L Stadon, S Waterson, H Welch

North Middlesex Hospital NHS Trust

B Jayaraman (PI), T Light

Nottingham University Hospitals NHS Trust & University of Nottingham

C E Bolton (PI), P Almeida, J Bonnington, M Chrystal, C Dupont, P Greenhaff, A Gupta, L Howard, W Jang, S Linford, L Matthews, R Needham, A Nikolaidis, S Prosper, K Shaw, A K Thomas

Oxford University Hospitals NHS Foundation Trust & University of Oxford

L P Ho (PI), N M Rahman (PI), M Ainsworth, A Alamoudi, A Bates, A Bloss, A Burns, P Carter, J Chen, F Conneh, T Dong, R I Evans, E Fraser, X Fu, J R Geddes, F Gleeson, P Harrison, M Havinden-Williams, P Jezzard, N Kanellakis, I Koychev, P Kurupati, X Li, H McShane, C Megson, K Motohashi, S Neubauer, D Nicoll, G Ogg, E Pacpaco, M Pavlides, Y Peng, N Petousi, N Rahman, B Raman, M J Rowland, K Saunders, M Sharpe, N Talbot, E Tunnicliffe

Royal Brompton and Harefield Clinical Group, Guy's and St Thomas' NHS Foundation Trust.

W D-C Man (PI), B Patel (PI), R E Barker, D Cristiano, N Dormand, M Gummadi, S Kon, K Liyanage, C M Nolan, S Patel, O Polgar, P Shah, S J Singh, J A Walsh

Royal Free London NHS Foundation Trust

J Hurst (PI), H Jarvis (PI), S Mandal (PI), S Ahmad, S Brill, L Lim, D Matila, O Olaosebikan, C Singh

Royal Papworth Hospital NHS Foundation Trust

M Toshner (PI), H Baxendale, L Garner, C Johnson, J Mackie, A Michael, J Pack, K Paques, H Parfrey, J Parmar

Salford Royal NHS Foundation Trust

N Diar Bakerly (PI), P Dark, D Evans, E Hardy, A Harvey, D Holgate, S Knight, N Mairs, N Majeed, L McMorrow, J Oxton, J Pendlebury, C Summersgill, R Ugwuoke, S Whittaker

Salisbury NHS Foundation Trust

W Matimba-Mupaya (PI), S Strong-Sheldrake

Sheffield Teaching NHS Foundation Trust & University of Sheffield

S L Rowland-Jones (PI), A A R Thompson (Co PI), J Bagshaw, M Begum, K Birchall, R Butcher, H Carborn, F Chan, K Chapman, Y Cheng, L Chetham, C Clark, Z Coburn, J Cole, M Dixon, A Fairman, J Finnigan, H Foot, D Foote, A Ford, R Gregory, K Harrington, L Haslam, L Hesselden, J Hockridge, A Holbourn, B Holroyd-Hind, L Holt, A Howell, E Hurditch, F Ilyas, C Jarman, A Lawrie, E Lee, J-H Lee, R Lenagh, A Lye, I Macharia, M Marshall, A Mbuyisa, J McNeill, S Megson, J Meiring, L Milner, S Misra, H Newell, T Newman, C Norman, L Nwafor, D Pattenadk, M Plowright, J Porter, P Ravencroft, C Roddis, J Rodger, P Saunders, J Sidebottom, J Smith, L Smith, N Steele, G Stephens, R Stimpson, B Thamu, N Tinker, K Turner, H Turton, P Wade, S Walker, J Watson, I Wilson, A Zawia

St George's University Hospitals NHS Foundation Trust

R Aul (PI), M Ali, A Dunleavy (PI), D Forton, N Msimanga, M Mencias, T Samakomva, S Siddique, J Teixeira, V Tavoukjian

Sherwood Forest Hospitals NHS Foundation Trust

J Hutchinson (PI), L Allsop, K Bennett, P Buckley, M Flynn, M Gill, C Goodwin, M Greatorex, H Gregory, C Heeley, L Holloway, M Holmes, J Kirk, W Lovegrove, TA Sewell, S Shelton, D Sissons, K Slack, S Smith, D Sowter, S Turner, V Whitworth, I Wynter

Shropshire Community Health NHS Trust

L Warburton (PI), S Painter, J Tomlinson

Somerset NHS Foundation Trust

C Vickers (PI), T Wainwright, D Redwood, J Tilley, S Palmer

Swansea Bay University Health Board

G A Davies (PI), L Connor, A Cook, T Rees, F Thaivalappil, C Thomas

Tameside and Glossop Integrated Care NHS Foundation

A Butt (PI), M Coulding, H Jones, S Kilroy, J McCormick, J McIntosh, H Savill, V Turner, J Vere

The Great Western Hospital Foundation Trust

E Fraile (PI), J Ugoji

The Hillingdon Hospitals NHS Foundation Trust

S S Kon (PI), H Lota, G Landers, M Nasseri, S Portukhay

The Rotherham NHS Foundation Trust

A Hormis (PI), A Daniels, J Ingham, L Zeidan

United Lincolnshire Hospitals NHS Trust

M Chablani (PI), L Osborne

University College London Hospital & University College London

M Marks (PI), J S Brown (PI), N Ahwireng, B Bang, D Basire, R C Chambers, A Checkley, R Evans, M Heightman, T Hillman, J Hurst, J Jacob, S Janes, R Jastrub, M Lipman, S Logan, D Lomas, M Merida Morillas, H Plant, J C Porter, K Roy, E Wall

University Hospital Birmingham NHS Foundation Trust & University of Birmingham

D Parekh (PI), N Ahmad Haider, C Atkin, R Baggott, M Bates, A Botkai, A Casey, B Cooper, J Dasgin, K Draxlbauer, N Gautam, J Hazeldine, T Hiwot, S Holden, K Isaacs, T Jackson, S Johnson, V Kamwa, D Lewis, J M Lord, S Madathil, C McGhee, K Mcgee, A Neal, A Newton Cox, J Nyaboko, D Parekh, Z Peterkin, H Qureshi, L Ratcliffe, E Sapey, J Short, T Soulsby, J Stockley, Z Suleiman, T Thompson, M Ventura, S Walder, C Welch, D Wilson, S Yasmin, K P Yip

University Hospitals of Derby and Burton

P Beckett (PI) C Dickens, U Nanda

University Hospitals of Leicester NHS Trust & University of Leicester

C E Brightling (CI), R A Evans (PI), M Aljaroof, N Armstrong, H Arnold, H Aung, M Bakali, M Bakau, M Baldwin, M Bingham, M Bourne, C Bourne, N Brunskill, P Cairns, L Carr, A Charalambou, C Christie, M J

Davies, S Diver, S Edwards, C Edwardson, O Elneima, H Evans, J Finch, S Glover, N Goodman, B Gootpu, N J Greening, K Hadley, P Haldar, B Hargadon, V C Harris, L Houchen-Wolloff, W Ibrahim, L Ingram, K Khunti, A Lea, D Lee, G P McCann, H J C McAuley, P McCourt, T Mcnally, A Moss, W Monteiro, M Pareek, S Parker, A Rowland, A Prickett, I N Qureshi, R Russell, M Sereno, A Shikotra, S Siddiqui, A Singapuri, S J Singh, J Skeemer, M Soares, E Stringer, T Thornton, M Tobin, L V Wain, T J C Ward, F Woodhead, T Yates, A Yousuf

University Hospital Southampton NHS Foundation Trust & University of Southampton

M G Jones (PI), C Childs, R Djukanovic, S Fletcher, M Harvey, E Marouzet, B Marshall, R Samuel, T Sass, T Wallis, H Wheeler

Whittington Health NHS

R Dharmagunawardena (PI), E Bright, P Crisp, M Stern

Wirral University Teaching Hospital

A Wight (PI), L Bailey, A Reddington

Wrightington Wigan and Leigh NHS trust

A Ashish (PI), J Cooper, E Robinson

Yeovil District Hospital NHS Foundation Trust

A Broadley (PI)

York & Scarborough NHS Foundation Trust

K Howard (PI), L Barman, C Brookes, K Elliott. L Griffiths, Z Guy, D Ionita, H Redfearn, C Sarginson
A Turnbull

Health and Care Research Wales

Y Ellis

London School of Hygiene & Tropical Medicine (LSHTM)

M Marks, A Briggs

NIHR Office for Clinical Research Infrastructure

K Holmes

Patient Public Involvement Leads

Asthma UK and British Lung Foundation Partnership - K Poinasamy, S Walker

Royal Surrey NHS Foundation Trust

M Halling-Brown

South London and Maudsley NHS Foundation Trust & Kings College London

G Breen, M Hotopf

Swansea University & Swansea Welsh Network

K Lewis, N Williams

Supplementary methods

Variables used in the analyses

Here we provide details on how the different variables used in the analyses were measured and encoded.

The following variables were used as markers of COVID-19 severity:

- WHO score: this is a scale defined by the WHO to capture the level of respiratory support needed by patients with COVID-19. It consists of 4 levels: no oxygen required (level 0), supplemental oxygen required (level 1), ventilation required (level 2, which we captured based on either continuous positive airway pressure ventilation, bi-level non-invasive ventilation or high-flow nasal oxygen needed at any point during hospital admission), and lastly invasive ventilation/oxygenation required (level 3, which was captured as either invasive mechanical ventilation or extra-corporeal membrane oxygenation). This was encoded as a continuous variable.
- National Early Warning Scores (NEWS): This scale captures the degree of departure of physical observations (a.k.a. vitals) from their normal range and is used nationally in the National Health Service (NHS) in the UK. It is a score ranging from 0 to 20 which we encoded as a continuous variable. Specifically, the following scoring is applied for the different physical observations and the total score is obtained by summing up the scores for the different items:
    - Respiratory Rate [breaths/min]: ≤ 8 (+2 points), 9-11 (+1 point), 12-20 (0 points), 21-21 (+2 points), ≥ 25 (+ 3 points);
    - Oxygen saturations: ≤ 91% (+3 points), 92-93% (+2 points), 94-95% (+1 point), ≥ 96% (+0 points);
    - Any supplemental oxygen: No (+0 points), Yes (+2 points);

- Temperature: ≤ 35°C (+3 points), 35.1-36°C (+1 point), 36.1-38°C (+0 points), 38.1-39°C (+1 point), ≥ 39.1°C (+2 points);
- Systolic Blood Pressure [mmHg]: ≤ 90 (+3 points), 91-100 (+2 points), 101-110 (+1 point), 111-219 (+0 points), ≥220 (+3 points);
- Heart Rate [beats per minute]: ≤ 40 (+3 points), 41-50 (+1 point), 51-90 (+0 points), 91-110 (+1 point), 111-130 (+2 points), ≥131 (+3 points).

- Recovery clusters: identified in a previous study based on the PHOSP cohort with clusters calculated using patient symptom questionnaires, physical performance, and cognitive assessment data.[1] The four resulting clusters, stratifying patients in terms of the severity of their recovery and the level of subsequent impairment, were categorised as follows:
  - Mild impairment;
  - Moderate impairment with cognitive impairment;
  - Severe impairment;
  - Very severe impairment.

  Recovery cluster variable was encoded categorically with mild impairment used as a reference level.
- Duration of hospital admission: captured from the participant's health record and recorded as a continuous variable
- Diagnosis of pulmonary embolism during hospitalisation: captured from the participant's health record and recorded as a continuous variable
- Admission to intensive care: captured from the participant's health record and recorded as a continuous variable
- Presence of altered consciousness or confusion during admission: captured from the participant's health record and recorded as a continuous variable

The neuropsychiatric outcomes used were the total Montreal Cognitive Assessment (MoCA), the Patient Health Questionnaire (PHQ-9), the Generalized Anxiety Disorder scale (GAD-7), and a cognitive subset of the Patient Symptom Questionnaire (PSQ). The latter is a score ranging from 0 to 7 which we encoded as a continuous variable. The latter was defined as the sum of the following items from PSQ:

- Within the last seven days, have you had any of these symptoms…?
    - Confusion (Yes: +1 point, No: 0 point)
    - Short term memory loss (Yes: +1 point, No: 0 point)
    - Difficulty with concentration (Yes: +1 point, No: 0 point)
    - Difficulty with communication (Yes: +1 point, No: 0 point)
    - Slowing down in your thinking (Yes: +1 point, No: 0 point)
- Do you have difficulty remembering or concentrating?:
    - No: 0 points
    - Yes, some difficulty: + 1/3 point
    - Yes, a lot of difficulty: + 2/3 points
    - Yes, cannot do at all: +1 point
- Do you have difficulty communicating, for example understanding or being understood?
    - No: 0 points
    - Yes, some difficulty: + 1/3 point
    - Yes, a lot of difficulty: + 2/3 points
    - Yes, cannot do at all: +1 point

The different covariates used in the analysis and their measurement were as follows:

- Diagnoses of respiratory condition
- Diagnosis of a rheumatological condition
- Diagnosis of cardiovascular condition
- Diagnosis of gastrointestinal condition
- History of a cerebrovascular accident
- Diagnosis of dementia
- Diagnosis of Parkinson's disease
- Diagnosis of depression or anxiety
- Diagnosis of another chronic condition, specifically chronic fatigue syndrome or chronic pain
- Diagnosis of diabetes
- Diagnosis of hypothyroidism/hyperthyroidism or other chronic metabolic/endocrine disorder
- Diagnosis of chronic kidney disease
- History of cancer
- History of chronic infectious diseases
- Educational level (highest level completed): encoded as a categorical variable with the following 8 categories:
    - None
    - Primary School
    - Secondary School (GCSE level, NVQ level ½ or equivalent, typically at the age of 16)
    - Sixth form college (A-levels, NVQ level 3 or equivalent, typically at the age of 18)
    - Vocational qualification (NVQ level 4 or equivalent)

- Undergraduate university degree or NVQ level 5 or equivalent
  - Post-graduate qualification
  - Prefer not to say
- Annual Household Income: encoded as a categorical variable with the following categories:
  - < £19,000
  - £19,001- £26,000
  - £26,001-£35,000
  - £35,001-£48,000
  - >£48,001
  - Prefer not to say
- Marital status: encoded with a single binary variable (married vs. not)
- Whether English was a patient's first language, as reported by the patient and encoded as a binary variable.

Details on statistical and robustness analyses

In this paper, we addressed two objectives:

- Objective 1: seeking association between markers of nervous system injury and post-acute neuropsychiatric outcomes
- Objective 2: seeking associations between markers of severity of the acute COVID-19 illness and markers of nervous system injury

In the primary analysis, only complete data was used (any participant with missing data on any of the exposure, outcome, or covariate data was discarded) and 3x8 separate regression models were estimated for Objective 1 (one for each marker of nervous system injury and

each neuropsychiatric outcome) and 7x3 separate regression models were estimated for Objective 2 (one for each marker of COVID-19 severity and each marker of nervous system injury, where each model returned one coefficient except for the model related to recovery clusters which returned 3 coefficients, one for each level of the categorical variable).

For both Objectives 1 and 2, we then ran 2 robustness analysis:

1) Using a single model incorporating all independent variables: for Objective 1, this meant that all three markers of nervous system injury were incorporated as independent variable in the regression model and only 8 separate regressions (rather than 24) were estimated (one for each neuropsychiatric outcome). For Objective 2, this meant that all seven markers of COVID-19 severity were incorporated as independent variables in the regression model and only three separate regressions (rather than 21) were estimated (one for each marker of nervous system injury).
2) Imputing missing data: Missing data on markers of COVID-19 severity, post-acute neuropsychiatric outcomes (i.e. GAD-7, PHQ-9, MoCA, and C-PSQ) or confounders were imputed (markers of nervous system injury were not imputed and only participants with complete data on these biomarkers were included). Variables used to predict aforementioned missing data were demographics, COVID-19 severity markers and markers of nervous system injury. The imputation was achieved using multiple imputation by chain equation model with 20 chains and 5 iterations, using the mice package (version 3.14.0). To maximise the information available in the imputation, the whole PHOSP-COVID cohort (not just those with markers of nervous system injury) was used to impute the missing data.

Supplementary figures

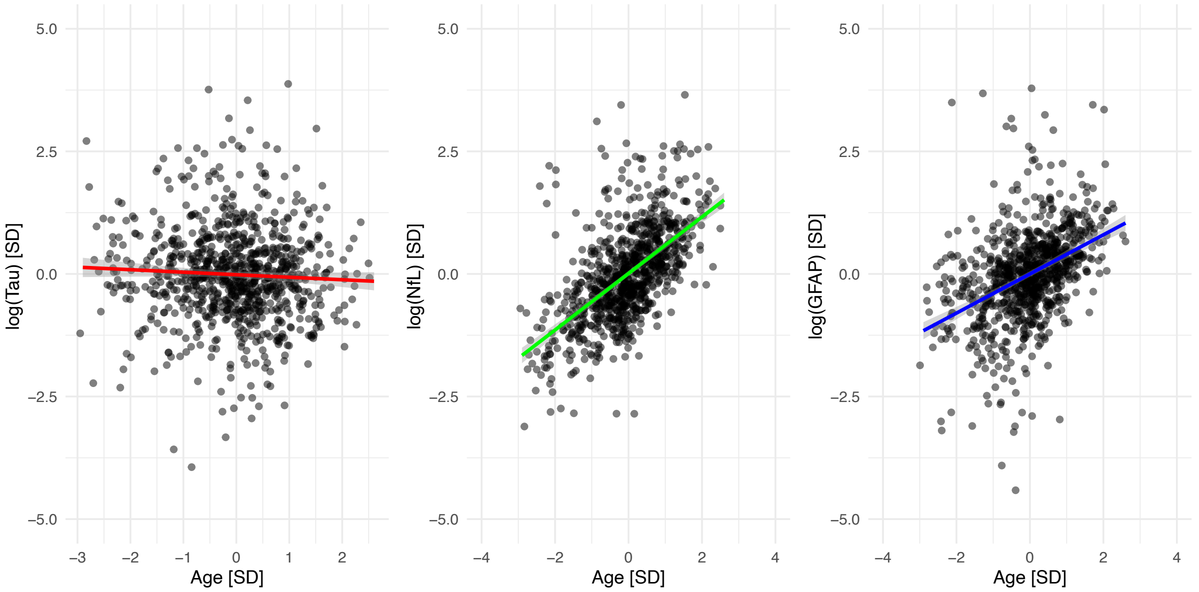

Supplementary Figure 1: Distributions of standardised markers of nervous system injury in logarithmic scale as functions of standardised age at hospitalisation admission. Pearson's correlation with standardised age is as follows; r (p-value): log(Tau) r = -0.05 (0.13); log(NfL) r = 0.57 (< 0.001); log(GFAP) r = 0.38 (< 0.001).

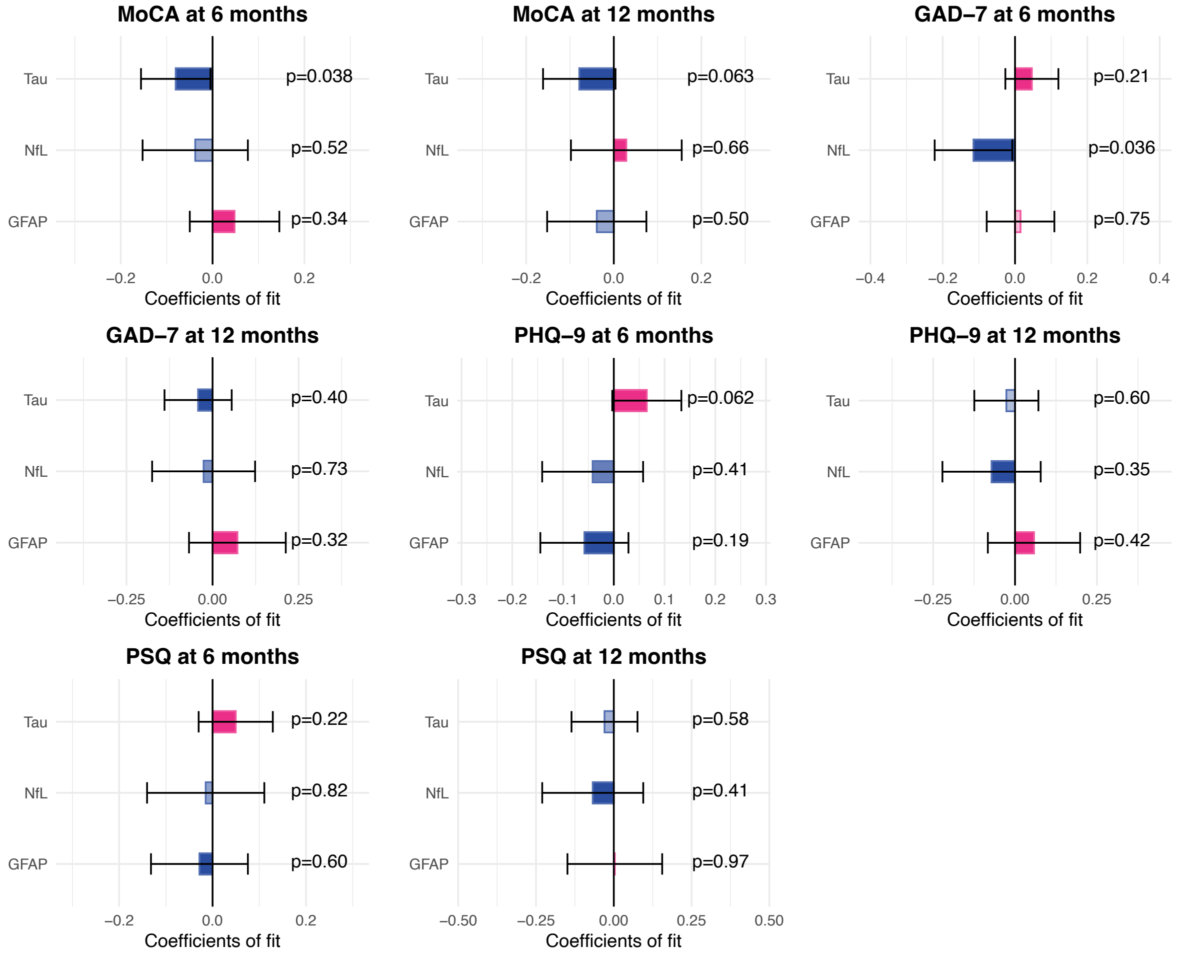

Supplementary Figure 2: Same as Figure 1 of the main manuscript but based on a model wherein all three markers of nervous system injury are included as independent variables in a single regression model. Uncorrected p-values are reported next to each bar and Bonferroni-corrected p-values meeting statistical significance thresholds are flagged as follows: * <0.05, **<0.01, ***<0.001

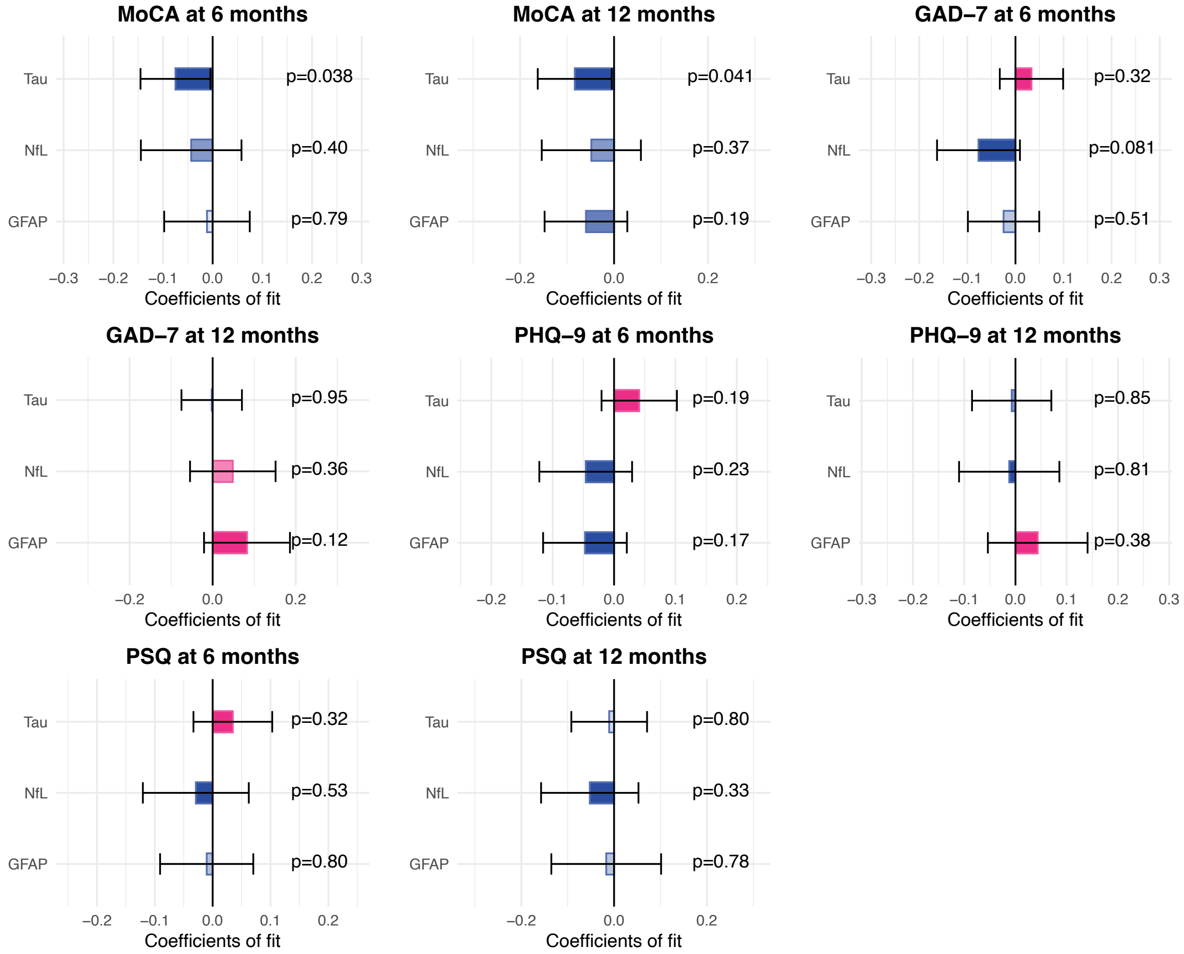

Supplementary Figure 3: Same as Figure 1 of the main manuscript but based on a dataset wherein missing data for covariates were imputed using multiple imputations and results pooled across imputations using Rubin's rule. Uncorrected p-values are reported next to each bar and Bonferroni-corrected p-values meeting statistical significance thresholds are flagged as follows: * <0.05, **<0.01, ***<0.001

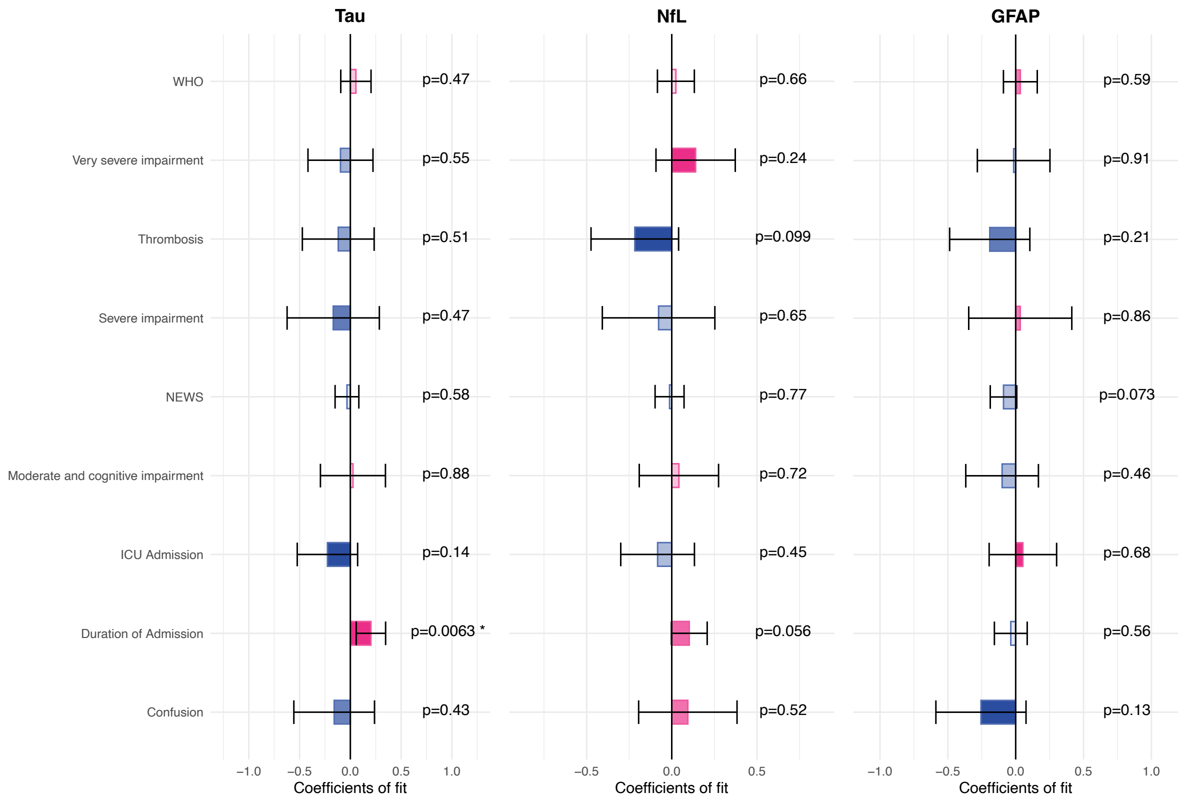

Supplementary Figure 4: Same as Figure 2 of the main manuscript but based on a model wherein all markers of severity of the acute COVID-19 illness are included as independent variables in a single regression model. Uncorrected p-values are reported next to each bar and Bonferroni-corrected p-values meeting statistical significance thresholds are flagged as follows: * <0.05, **<0.01, ***<0.001

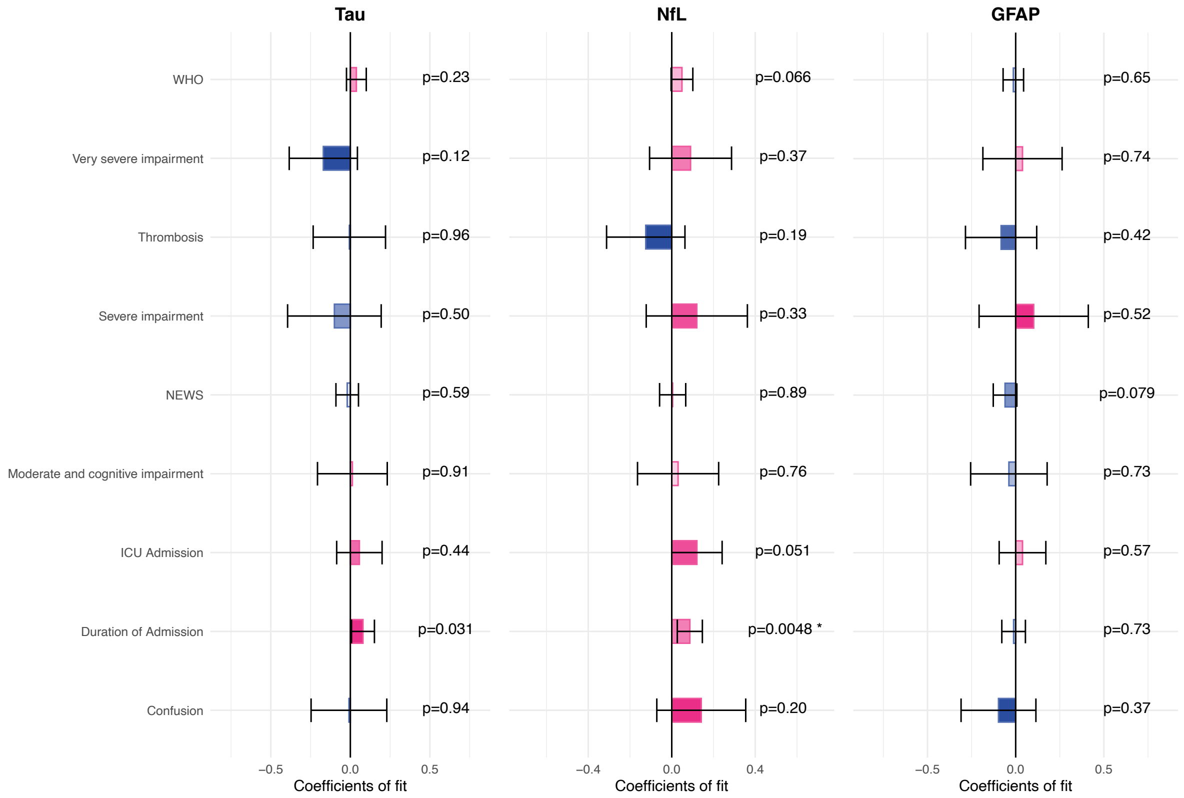

Supplementary Figure 5: Same as Figure 2 of the main manuscript but based on a dataset wherein missing data for covariates were imputed using multiple imputations and results pooled across imputations using Rubin's rule. Uncorrected p-values are reported next to each bar and Bonferroni-corrected p-values meeting statistical significance thresholds are flagged as follows: * <0.05, **<0.01, ***<0.001

Supplementary tables

Supplementary Table 1. Baseline characteristics for the cohort used in this study and for all other PHOSP-COVID participants.

**Cohort**	**PHOSP-COVID participants with neural injury markers**	**All other PHOSP-COVID participants**
COHORT SIZE	891	1806
SOCIODEMOGRAPHIC FACTORS		
Age; mean (SD)	57.3 (12.5)	58.2 (12.8)
Sex; n (%)		
Female	312 (35.0)	637 (35.3)
Male	535 (60.0)	958 (53.0)
Unknown	44 (4.9)	211 (11.7)
Race; n (%)		
White	668 (75.0)	1199 (66.4)
Asian	122 (13.7)	188 (10.4)
Black	47 (5.3)	130 (7.2)
Mixed	14 (1.6)	37 (2.0)
Other	24 (2.7)	75 (4.2)
Unknown	16 (1.8)	177 (9.8)
Educational level; n (%)		
Primary school	16 (1.8)	41 (2.3)
Secondary school	271 (30.4)	488 (27.0)
Sixth form college	105 (11.8)	212 (11.7)
Vocational Qualification	97 (10.9)	192 (10.6)
Undergraduate university degree	157 (17.6)	279 (15.4)
Post-graduate qualification	113 (12.7)	232 (12.8)
None	23 (2.6)	45 (2.5)
Prefer not to say	29 (3.3)	57 (3.2)
Unknown	80 (9.0)	260 (14.4)
Household income; n (%)		
< £19,000	104 (11.7)	275 (15.2)
£19,001 – £26,000	100 (11.2)	195 (10.8)
£26,001 – £35,000	104 (11.7)	180 (10.0)

£35,001 – £48,000	105 (11.8)	180 (10.0)
>£48,001	210 (23.6)	366 (20.3)
Prefer not to say	189 (21.2)	346 (19.2)
Unknown	79 (8.9)	264 (14.6)
Marital status; n (%)		
Married	500 (56.1)	895 (49.6)
Unmarried	322 (36.1)	662 (36.7)
Unknown	69 (7.7)	249 (13.8)
English as a first language; n (%)		
Yes	691 (77.6)	1298 (71.9)
No	135 (15.2)	271 (15.0)
Unknown	65 (7.3)	237 (13.1)
COMORBIDITIES; n (%)		
Cancer	61 (6.8)	122 (6.8)
Cardiovascular condition	372 (41.8)	791 (43.8)
ME/CFS/Fibromyalgia/Chronic pain	40 (4.5)	91 (5.0)
Chronic kidney disease	33 (3.7)	73 (4.0)
Cerebrovascular accident	33 (3.7)	70 (3.9)
Dementia	< 10	< 10
Diabetes	166 (18.6)	351 (19.4)
Metabolic/endocrine disorder	65 (7.3)	152 (8.4)
Gastrointestinal condition	179 (20.1)	371 (20.5)
Infectious disease	21 (2.4)	49 (2.7)
Parkinson’s disease	< 10	< 10
Depression or anxiety	151 (16.9)	308 (17.1)
Respiratory condition	254 (28.5)	457 (25.3)
Rheumatological condition	121 (13.6)	273 (15.1)

Supplementary Table 2: Baseline characteristics of the cohort with a 12-month follow-up

**Cohort**	
COHORT SIZE	507
SOCIODEMOGRAPHIC FACTORS	
Age; mean (SD)	58.8 (12.0)
Sex; n (%)	
Female	170 (33.5)
Male	320 (63.1)
Unknown	17 (3.4)
Ethnicity; n (%)	
White	396 (78.1)
Asian	66 (13.0)
Black	22 (4.3)
Mixed	8 (1.6)
Other	12 (2.4)
Unknown	3 (0.6)
Educational level; n (%)	
Primary school	6 (1.2)
Secondary school	154 (30.4)
Sixth form college	61 (12.0)
Vocational qualification	61 (12.0)
Undergraduate university degree	92 (18.1)
Post-graduate qualification	67 (13.2)
None	15 (3.0)
Prefer not to say	13 (2.6)
Unknown	38 (7.5)
Household income; n (%)	
< £19,000	58 (11.4)
£19,001 – £26,000	57 (11.2)
£26,001 – £35,000	66 (13.0)
£35,001 – £48,000	60 (11.8)
>£48,001	118 (23.3)
Prefer not to say	112 (22.1)
Unknown	36 (7.1)
Marital status; n (%)	
Married	298 (58.8)
Unmarried	179 (35.3)
Unknown	30 (5.9)
English as a first language; n (%)	
Yes	407 (80.3)
No	75 (14.8)
Unknown	25 (4.9)
COMORBIDITIES; n (%)	
Cancer	34 (6.7)
Cardiovascular condition	230 (45.4)
ME/CFS/Fibromyalgia/Chronic pain	22 (4.3)
Chronic kidney disease	17 (3.4)

Cerebrovascular accident	21 (4.1)
Dementia	< 10
Diabetes	107 (21.1)
Metabolic/endocrine disorder	40 (7.9)
Gastrointestinal condition	98 (19.3)
Infectious disease	11 (2.2)
Parkinson's disease	< 10
Depression or anxiety	83 (16.4)
Respiratory condition	143 (28.2)
Rheumatological condition	63 (12.4)
COVID-19 SEVERITY MARKERS	
WHO; mean (SD)	2.53 (1.09)
NEWS; mean (SD)	3.76 (2.44)
Duration of hospital admission; mean (SD)	16.5 (20.9)
Pulmonary embolism; n (%)	52 (10.3)
Altered consciousness/confusion; n (%)	48 (9.5)
COVID-19 Recovery clusters; n (%)	
Mild impairment	69 (13.6)
Moderate and cognitive impairment	111 (21.9)
Severe impairment	49 (9.7)
Very severe impairment	177 (34.9)
Unknown	101 (19.9)
CLINICAL SCALES AT 6 MONTHS; mean (SD)	
MoCA	26.4 (3.21)
GAD-7	5.02 (5.83)
PHQ-9	6.34 (6.62)
PSQ	2.05 (2.11)
NEURAL INJURY MARKERS; mean (SD)	
GFAP	4.30 (0.85)
Tau protein	1.72 (0.79)
NfL	2.29 (0.69)

Supplementary Table 3: Regression coefficients and 95% confidence intervals for the variables (covariates and neural injury markers) used in the regression model wherein clinical scales measured at 6 months in each column are dependent variables.

	Dependent variables			
	**MoCA at 6 months**	**GAD-7 at 6 months**	**PHQ-9 at 6 months**	**C-PSQ at 6 months**
**Covariates**				
Cardiovascular condition	0.083 (-0.084 - 0.25)	0.22 (0.058 - 0.38)	0.16 (0.013 - 0.31)	0.05 (-0.13 – 0.23)
Cerebrovascular accident	-0.41 (-0.79 - -0.026)	-0.11 (-0.45 - 0.24)	-0.029 (-0.35 - 0.29)	0.065 (-0.32 – 0.45)
Dementia	1.13 (-0.71 - 2.96)	0.55 (-1.31 - 2.41)	0.13 (-1.60 - 1.86)	0.71 (-1.16 – 2.59)
Parkinson's disease	0.31 (-1.47 - 2.09)	0.20 (-1.09 - 1.50)	0.62 (-0.58 - 1.82)	-0.55 (-2.39 – 1.29)
Depression or anxiety	-0.12 (-0.33 - 0.084)	0.67 (0.47 - 0.87)	0.62 (0.44 - 0.81)	0.39 (0.17 – 0.61)
ME/CFS/Fibromyalgia/Chronic pain	-0.48 (-0.84 - -0.12)	0.66 (0.31 - 1.01)	0.57 (0.25 - 0.89)	0.49 (0.13 – 0.85)
Diabetes	-0.14 (-0.33 - 0.053)	0.12 (-0.065 - 0.31)	0.058 (-0.12 - 0.23)	0.045 (-0.17 – 0.26)
Respiratory condition	-0.0093 (-0.17 - 0.15)	0.14 (-0.019 - 0.29)	0.083 (-0.059 - 0.23)	-0.0079 (-0.18 – 0.16)
Rheumatological condition	0.18 (-0.039 - 0.40)	0.094 (-0.12 - 0.30)	0.21 (0.012 - 0.40)	0.068 (-0.16 – 0.29)
Gastrointestinal condition	0.012 (-0.17 - 0.19)	0.021 (-0.15 - 0.19)	0.089 (-0.072 - 0.25)	-0.081 (-0.27 – 0.11)
Metabolic/endocrine disorder	-0.036 (-0.31 - 0.24)	0.04 (-0.22 - 0.30)	-0.044 (-0.29 - 0.20)	0.14 (-0.14 – 0.41)
Chronic kidney disease	-0.13 (-0.50 - 0.24)	-0.072 (-0.43 - 0.28)	-0.013 (-0.34 - 0.32)	0.059 (-0.33 – 0.45)
Cancer	-0.17 (-0.44 - 0.11)	0.17 (-0.10 - 0.44)	0.14 (-0.11 - 0.39)	-0.076 (-0.33 – 0.45)
Chronis infectious disease	0.11 (-0.40 - 0.62)	-0.50 (-0.95 - -0.06)	-0.26 (-0.67 - 0.15)	-0.12 (-0.62 – 0.39)
Age	-0.22 (-0.33 - -0.12)	-0.097 (-0.20 - 0.0021)	-0.14 (-0.23 - -0.049)	-0.10 (-0.21 – 0.013)
Sex (female)	-0.06 (-0.22 - 0.097)	0.12 (-0.031 - 0.27)	0.16 (0.023 - 0.31)	0.055 (-0.12 – 0.23)
Race (reference: White)				
Mixed	-0.40 (-1.01 - 0.21)	0.078 (-0.51 - 0.67)	-0.29 (-0.84 - 0.26)	-0.063 (-0.66 – 0.54)
Asian	-0.064 (-0.34 - 0.21)	-0.21 (-0.48 - 0.063)	-0.15 (-0.40 - 0.094)	-0.24 (-0.54 – 0.063)
Black	-0.64 (-0.97 - -0.31)	0.012 (-0.32 - 0.34)	-0.14 (-0.45 - 0.17)	-0.18 (-0.57 – 0.21)
Other	-0.17 (-0.67 - 0.34)	-0.081 (-0.58 - 0.42)	-0.0092 (-0.47 - 0.45)	-0.32 (-0.84 – 0.19)
Educational level (reference: None)				
Primary school	-0.67 (-1.37 - 0.027)	-0.19 (-0.85 - 0.47)	-0.36 (-0.97 - 0.26)	-0.37 (-1.07 – 0.33)
Secondary school	0.50 (0.03 - 0.97)	-0.16 (-0.62 - 0.31)	-0.049 (-0.48 - 0.38)	-0.03 (-0.55 – 0.49)
Sixth form college	0.65 (0.16 - 1.15)	-0.11 (-0.61 - 0.38)	-0.057 (-0.51 - 0.40)	-0.083 (-0.63 – 0.46)
Vocational qualification	0.71 (0.21 - 1.21)	0.017 (-0.47 - 0.51)	0.099 (-0.36 - 0.55)	0.24 (-0.30 – 0.46)
Undergraduate university degree	0.65 (0.15 - 1.14)	-0.18 (-0.67 - 0.30)	-0.20 (-0.65 - 0.24)	0.10 (-0.43 – 0.64)
Post-graduate qualification	0.99 (0.48 - 1.50)	-0.26 (-0.76 - 0.24)	-0.14 (-0.61 - 0.32)	-0.008 (-0.57 – 0.55)
Prefer not to say	0.72 (0.13 - 1.31)	0.11 (-0.46 - 0.68)	0.10 (-0.42 - 0.63)	0.70 (0.048 – 1.36)
Income (reference: < £19,000)				
£19,001 – £26,000	-0.024 (-0.32 - 0.27)	-0.45 (-0.74 - -0.17)	-0.53 (-0.79 - -0.26)	-0.18 (-0.49 – 0.12)
£26,001 – £35,000	0.18 (-0.11 - 0.47)	-0.66 (-0.94 - -0.39)	-0.68 (-0.94 - -0.42)	-0.39 (-0.69 - -0.087)
£35,001 – £48,000	0.12 (-0.18 - 0.42)	-0.44 (-0.73 - -0.15)	-0.52 (-0.79 - -0.25)	-0.38 (-0.71 - -0.06)
>£48,001	0.30 (0.022 - 0.57)	-0.49 (-0.75 - -0.22)	-0.56 (-0.80 - -0.31)	-0.32 (-0.60 - -0.026)

Prefer not to say	0.12 (-0.14 - 0.37)	-0.26 (-0.52 - -0.014)	-0.41 (-0.64 - -0.17)	-0.29 (-0.56 - -0.019)
Is Married	0.11 (-0.046 - 0.26)	-0.15 (-0.30 - -0.0043)	-0.19 (-0.33 - -0.053)	-0.067 (-0.23 – 0.099)
English as a first language	0.54 (0.27 - 0.81)	-0.35 (-0.60 - -0.091)	-0.064 (-0.30 - 0.17)	0.11 (-0.17 – 0.40)
**Neural injury markers**				
Tau	-0.08 (-0.16 - -0.0047)	0.047 (-0.027 - 0.12)	0.065 (-0.0031 - 0.13)	0.05 (-0.03 – 0.13)
NfL	-0.038 (-0.15 - 0.077)	-0.12 (-0.22 - -0.0076)	-0.042 (-0.14 - 0.058)	-0.015 (-0.14 – 0.11)
GFAP	0.048 (-0.05 - 0.15)	0.015 (-0.079 - 0.11)	-0.058 (-0.15 - 0.029)	-0.028 (-0.13 – 0.0076)

Supplementary Table 4: Regression coefficients and 95% confidence intervals for the variables (covariates and neural injury markers) used in the regression model wherein clinical scales measured at 12 months in each column are dependent variables.

	**Dependent variables**			
	**MoCA at 12 months**	**GAD-7 at 12 months**	**PHQ-9 at 12 months**	**C-PSQ at 12 months**
**Covariates**				
Cardiovascular condition	0.03 (-0.16 - 0.22)	0.25 (0.023 - 0.47)	0.13 (-0.096 - 0.36)	0.22 (-0.03 – 0.46)
Cerebrovascular accident	-0.17 (-0.55 - 0.21)	-0.26 (-0.72 - 0.20)	-0.13 (-0.60 - 0.35)	0.086 (-0.43 – 0.61)
Dementia	0.76 (-0.85 - 2.38)	1.21 (-0.75 - 3.18)	0.33 (-1.65 - 2.30)	0.67 (-1.37 – 2.71)
Parkinson's disease	0.42 (-0.68 - 1.51)	0.95 (-0.39 - 2.29)	0.36 (-0.98 - 1.70)	0.45 (-0.94 – 1.83)
Depression or anxiety	-0.11 (-0.35 - 0.13)	0.65 (0.36 - 0.93)	0.64 (0.36 - 0.92)	0.54 (0.24 – 0.84)
ME/CFS/Fibromyalgia/ Chronic pain	-0.57 (-1.00 - -0.14)	0.63 (0.11 - 1.15)	0.68 (0.16 - 1.20)	0.84 (0.26 – 1.42)
Diabetes	-0.29 (-0.50 - -0.079)	0.02 (-0.23 - 0.27)	0.067 (-0.19 - 0.32)	0.17 (-0.11 – 0.45)
Respiratory condition	0.10 (-0.073 - 0.28)	-0.074 (-0.29 - 0.14)	-0.08 (-0.29 - 0.13)	-0.046 (-0.28 – 0.19)
Rheumatological condition	0.00068 (-0.25 - 0.25)	-0.036 (-0.34 - 0.27)	0.10 (-0.20 - 0.41)	-0.058 (-0.38 – 0.26)
Gastrointestinal condition	0.15 (-0.059 - 0.36)	0.067 (-0.19 - 0.32)	0.068 (-0.19 - 0.33)	-0.092 (-0.37 – 0.18)
Metabolic/endocrine disorder	-0.13 (-0.45 - 0.20)	0.03 (-0.33 - 0.39)	0.026 (-0.34 - 0.39)	0.051 (-0.36 – 0.46)
Chronic kidney disease	0.065 (-0.34 - 0.47)	0.15 (-0.35 - 0.64)	-0.041 (-0.54 - 0.46)	0.055 (-0.50 – 0.61)
Cancer	0.0057 (-0.32 - 0.33)	0.087 (-0.31 - 0.49)	-0.06 (-0.46 - 0.34)	0.11 (-0.32 – 0.55)
Chronic infectious disease	-0.73 (-1.32 - -0.13)	-0.086 (-0.69 - 0.51)	-0.16 (-0.76 - 0.44)	-0.069 (-0.69 – 0.55)
Age	-0.25 (-0.37 - -0.12)	-0.21 (-0.36 - -0.068)	-0.17 (-0.32 - -0.024)	-0.18 (-0.34 - -0.016)
Sex (female)	0.097 (-0.081 - 0.27)	0.15 (-0.061 - 0.35)	0.10 (-0.11 - 0.31)	0.032 (-0.20 – 0.26)
Race (Reference: White)				
Mixed	-1.30 (-2.08 - -0.52)	0.031 (-0.76 - 0.82)	-0.20 (-0.99 - 0.59)	0.056 (-0.76 – 0.87)
Asian	-0.038 (-0.37 - 0.29)	0.21 (-0.16 - 0.58)	0.14 (-0.23 - 0.51)	-0.12 (-0.53 – 0.29)
Black	-0.67 (-1.10 - -0.24)	0.087 (-0.42 - 0.59)	-0.087 (-0.60 - 0.42)	-0.24 (-0.75 – 0.27)
Other	0.011 (-0.59 - 0.61)	-0.016 (-0.68 - 0.65)	-0.0099 (-0.68 - 0.66)	-0.14 (-0.90 – 0.61)
Educational level (reference: None)				
Primary school	-0.93 (-1.86 - -0.0063)	-0.27 (-1.35 - 0.82)	-0.38 (-1.47 - 0.71)	-0.62 (-1.93 – 0.68)
Secondary school	-0.0023 (-0.54 - 0.53)	-0.0093 (-0.68 - 0.66)	0.089 (-0.58 - 0.76)	-0.20 (-1.09 – 0.69)
Sixth form college	0.14 (-0.42 - 0.71)	-0.14 (-0.85 - 0.56)	-0.074 (-0.78 - 0.63)	-0.27 (-1.19 – 0.65)
Vocational qualification	0.15 (-0.42 - 0.71)	0.007 (-0.70 - 0.71)	0.16 (-0.55 - 0.87)	-0.047 (-0.97 – 0.87)
Undergraduate university degree	0.23 (-0.33 - 0.79)	-0.14 (-0.84 - 0.57)	0.041 (-0.66 - 0.74)	-0.15 (-1.07 – 0.76)
Post-graduate qualification	0.42 (-0.15 - 0.99)	-0.24 (-0.96 - 0.48)	-0.01 (-0.74 - 0.72)	-0.30 (-1.22 – 0.63)
Prefer not to say	-0.28 (-0.99 - 0.43)	0.16 (-0.68 - 0.99)	0.12 (-0.72 - 0.96)	0.15 (-0.89 – 1.18)
Income (Reference: < £19,000)				
£19,001 – £26,000	0.36 (0.019 - 0.70)	-0.31 (-0.71 - 0.094)	-0.32 (-0.73 - 0.085)	-0.24 (-0.70 – 0.23)
£26,001 – £35,000	0.52 (0.20 - 0.85)	-0.42 (-0.81 - -0.028)	-0.59 (-0.98 - -0.19)	-0.20 (-0.64 – 0.24)
£35,001 – £48,000	0.29 (-0.054 - 0.63)	-0.23 (-0.64 - 0.19)	-0.44 (-0.86 - -0.026)	-0.11 (-0.60 – 0.37)
>£48,001	0.58 (0.26 - 0.90)	-0.47 (-0.85 - -0.088)	-0.60 (-0.98 - -0.22)	-0.16 (-0.60 – 0.28)

Prefer not to say	0.54 (0.24 - 0.85)	0.0019 (-0.36 - 0.37)	-0.23 (-0.60 - 0.14)	-0.055 (-0.47 – 0.36)
Is Married	0.092 (-0.082 - 0.27)	-0.09 (-0.30 - 0.12)	-0.20 (-0.40 - 0.011)	-0.19 (-0.42 – 0.035)
English as a first language	0.64 (0.33 - 0.95)	0.11 (-0.24 - 0.46)	0.19 (-0.16 - 0.54)	0.11 (-0.27 – 0.49)
**Neural injury markers**				
Tau	-0.079 (-0.16 - 0.0039)	-0.042 (-0.14 - 0.056)	-0.026 (-0.12 - 0.071)	-0.03 (-0.14 – 0.076)
NfL	0.029 (-0.098 - 0.16)	-0.026 (-0.18 - 0.12)	-0.072 (-0.22 - 0.078)	-0.068 (-0.23 – 0.094)
GFAP	-0.039 (-0.15 - 0.074)	0.072 (-0.068 - 0.21)	0.058 (-0.083 - 0.20)	0.0025 (-0.15 – 0.15)

Supplementary Table 5: Results of the analysis of individual cognitive domains. Regression coefficients and 95% confidence intervals for the association between neural injury markers (independent variables) and individual domains of the Montreal Cognitive Assessment (MoCA; dependent variables) recorded at 6 months post-COVID-19.

	**Neural injury markers**					
	**GFAP**		**Tau**		**NfL**	
**Cognitive domain**	**Coefficient (95% CI)**	**P-value**	**Coefficient (95% CI)**	**P-value**	**Coefficient (95% CI)**	**P-value**
MoCA (visuospatial)	-0.068 (-0.15 - 0.017)	0.12	-0.066 (-0.14 - 0.0092)	0.086	-0.057 (-0.15 - 0.04)	0.25
MoCA (naming)	0.017 (-0.068 - 0.10)	0.7	-0.032 (-0.11 - 0.043)	0.4	-0.036 (-0.13 - 0.06)	0.47
MoCA (attention)	0.053 (-0.04 - 0.15)	0.26	-0.037 (-0.12 - 0.046)	0.38	0.033 (-0.073 - 0.14)	0.55
MoCA (language)	0.012 (-0.073 - 0.096)	0.79	-0.036 (-0.11 - 0.039)	0.35	-0.037 (-0.13 - 0.06)	0.45
MoCA (abstract)	-0.0089 (-0.099 - 0.081)	0.85	-0.036 (-0.12 - 0.044)	0.38	-0.021 (-0.12 - 0.081)	0.69
MoCA (recall)	-0.00051 (-0.085 - 0.084)	0.99	-0.066 (-0.14 - 0.0096)	0.088	-0.055 (-0.15 - 0.041)	0.26
MoCA (orientation)	0.059 (-0.033 - 0.15)	0.21	-0.024 (-0.11 - 0.058)	0.57	0.039 (-0.065 - 0.14)	0.47

References

1. Evans RA, McAuley H, Harrison EM, et al. Physical, cognitive, and mental health impacts of COVID-19 after hospitalisation (PHOSP-COVID): a UK multicentre, prospective cohort study. *Lancet Respir Med*. 2021;9(11):1275-1287.
